# THEMIS attenuates MASH by suppressing disease-associated hepatocyte induction and hepatocyte senescence in mice

**DOI:** 10.1172/JCI199303

**Published:** 2026-05-01

**Authors:** Xiaoxue Qiu, You Lu, Yuwei Tang, Linkang Zhou, Yu-tung Lee, Ziyi Meng, Zhimin Chen, Fnu Pradeepa, Lanuza A.P. Faccioli, Zhiping Hu, Alejandro Soto-Gutierrez, Siming Li, Jiandie D. Lin

**Affiliations:** 1 Life Sciences Institute and Department of Cell & Developmental Biology, University of Michigan Medical Center, Ann Arbor, Michigan, USA.; 2 Department of Pathology, University of Pittsburgh School of Medicine, Pittsburgh, Pennsylvania, USA.; 3 Center for Transcriptional Medicine, University of Pittsburgh, Pittsburgh, Pennsylvania, USA.

**Keywords:** Hepatology, Metabolism, Cellular senescence

## Abstract

Hepatocyte senescence is increasingly recognized as a pathogenic driver of metabolic dysfunction–associated steatohepatitis (MASH). Through single-nucleus transcriptomic profiling, we identified a discrete population of disease-associated hepatocytes (daHep) exhibiting enrichment for senescence markers in MASH livers. The emergence of senescent hepatocytes was associated with a marked induction of hepatic thymocyte selection associated (THEMIS) expression in both murine and human MASH. Genetic ablation of *Themis*, either globally or specifically in hepatocytes, resulted in significant expansion of daHep and senescent hepatocyte populations and exacerbated MASH pathology in mice. Single-nucleus transcriptomic analysis revealed a central role for THEMIS in shaping the cellular landscape of both parenchymal and nonparenchymal compartments within the MASH liver microenvironment. Conversely, adeno-associated virus–mediated overexpression of THEMIS suppressed hepatocyte senescence and attenuated diet-induced MASH. Mechanistic studies revealed that THEMIS deficiency promoted aberrant ERK phosphorylation and hepatocyte senescence. These findings establish THEMIS as a critical hepatoprotective factor that restrains hepatocyte senescence and mitigates metabolic liver disease progression.

## Abstract 2

Hepatic THEMIS protects against metabolic dysfunction–associated steatohepatitis development, through improving metabolic functions, ameliorating hepatocyte senescence and inhibition of disease‑associated hepatocytes induction.

## Introduction

Metabolic dysfunction–associated steatohepatitis (MASH) is the severe form of metabolic dysfunction–associated steatotic liver disease (MASLD), characterized by hepatic steatosis, liver injury, inflammation, and fibrosis (1–4). MASH pathogenesis involves complex interactions among hepatic metabolic derangements, hepatocyte cell death, and persistent inflammatory and fibrotic responses in the liver. Multiple intrinsic and extrinsic factors contribute to disease progression, including mitochondrial dysfunction, lipotoxicity, oxidative stress, endoplasmic reticulum stress, and gut-derived endotoxin exposure (5). These pathogenic stressors exacerbate hepatocyte injury and promote the release of danger-associated molecular patterns, such as ATP, mitochondria, and lipid droplets (LD) (6), which in turn trigger proinflammatory responses within the liver microenvironment. In response, hepatocytes activate diverse stress response mechanisms that facilitate the restoration of metabolic homeostasis and tissue function during MASH. However, this reprogramming of hepatic signaling and cellular functions can become maladaptive, leading to deleterious effects that further drive disease progression, such as aberrant activation of liver fibrosis, persistent inflammatory cytokine production, and hepatocyte senescence. The precise nature of the hepatoprotective adaptive response in hepatocytes during MASH pathogenesis remains largely unresolved.

Hepatocytes constitute approximately 60%–70% of the total cell population in the mammalian liver, while nonparenchymal cells (NPCs), including endothelial cells, Kupffer cells, hepatic stellate cells (HSCs), cholangiocytes, and diverse immune cell types, constitute the rest (7–11). Recent advances in single-cell transcriptomics have provided unprecedented insights into liver cell heterogeneity and cellular reprogramming during liver disease (12–14). Similar to hepatocytes, sinusoidal endothelial cells, liver macrophages, and HSCs exhibit spatial heterogeneity and zonation along the portal to central axis of the liver lobule (12, 15, 16). Several notable features of transcriptomic heterogeneity and cell state transitions have been observed during MASH progression. For example, HSCs transition from a quiescent phenotype in the healthy liver to activated myofibroblasts that are highly active in secreting and remodeling extracellular matrix, thereby contributing to liver fibrosis and scar formation (17–19).

Additionally, macrophages expressing *Trem2* are nearly absent in healthy liver but show marked induction in both mouse and human MASH (13, 14). These TREM2^+^ macrophages form crown-like structures in the MASH liver and play multifaceted roles in efferocytosis and NLRP3 inflammasome activation (20, 21). Recent work has revealed disease-associated hepatocytes (daHep) as a unique population that emerges during liver injury and tumorigenesis, illustrating hepatocyte heterogeneity beyond liver zonation (22, 23).

Cellular senescence is characterized by permanent cell cycle arrest and distinct molecular features, including upregulation of p21 (encoded by *Cdkn1a*) and p16INK4a (encoded by *Cdkn2a*) expression, increased senescence-associated β-galactosidase (SA-β-Gal) activity, and induction of the senescence-associated secretory phenotype (SASP) (24–27). The latter entails the secretion of proinflammatory cytokines, chemokines, growth factors, and matrix metalloproteinases that exert both autocrine and paracrine effects on the neighboring cells. Recent studies have implicated cellular senescence as a potential causative factor in metabolic disorders, including insulin resistance and MASLD (28–30). Clearance of senescent cells using small-molecule senolytics leads to improved physical and metabolic parameters in mice (31, 32). Adipose tissue senescence is markedly increased during aging and obesity, leading to impaired adipogenesis, reduced fat storage capacity, and dysregulated adipocyte metabolism and function (33, 34). Similarly, senescent hepatocytes contribute to liver dysfunction during aging, MASH progression, and the development of MASH-associated hepatocellular carcinoma (32, 35–38). In this context, the secreted factors BMP4 and Gremlin 1 modulate hepatocyte senescence through opposing effects on the YAP/TAZ pathway (35). Despite these findings, it remains unknown whether adaptive mechanisms are engaged to restrain hepatocyte senescence and protect against MASH pathogenesis. In this study, we identified thymocyte selection associated (THEMIS), a well-known factor regulating T cell development, as a hepatoprotective factor induced in the MASH liver that suppresses hepatocyte senescence and disease progression.

## Results

### Emergence of deHep with senescence hallmarks in MASH liver.

**Figure 1:**
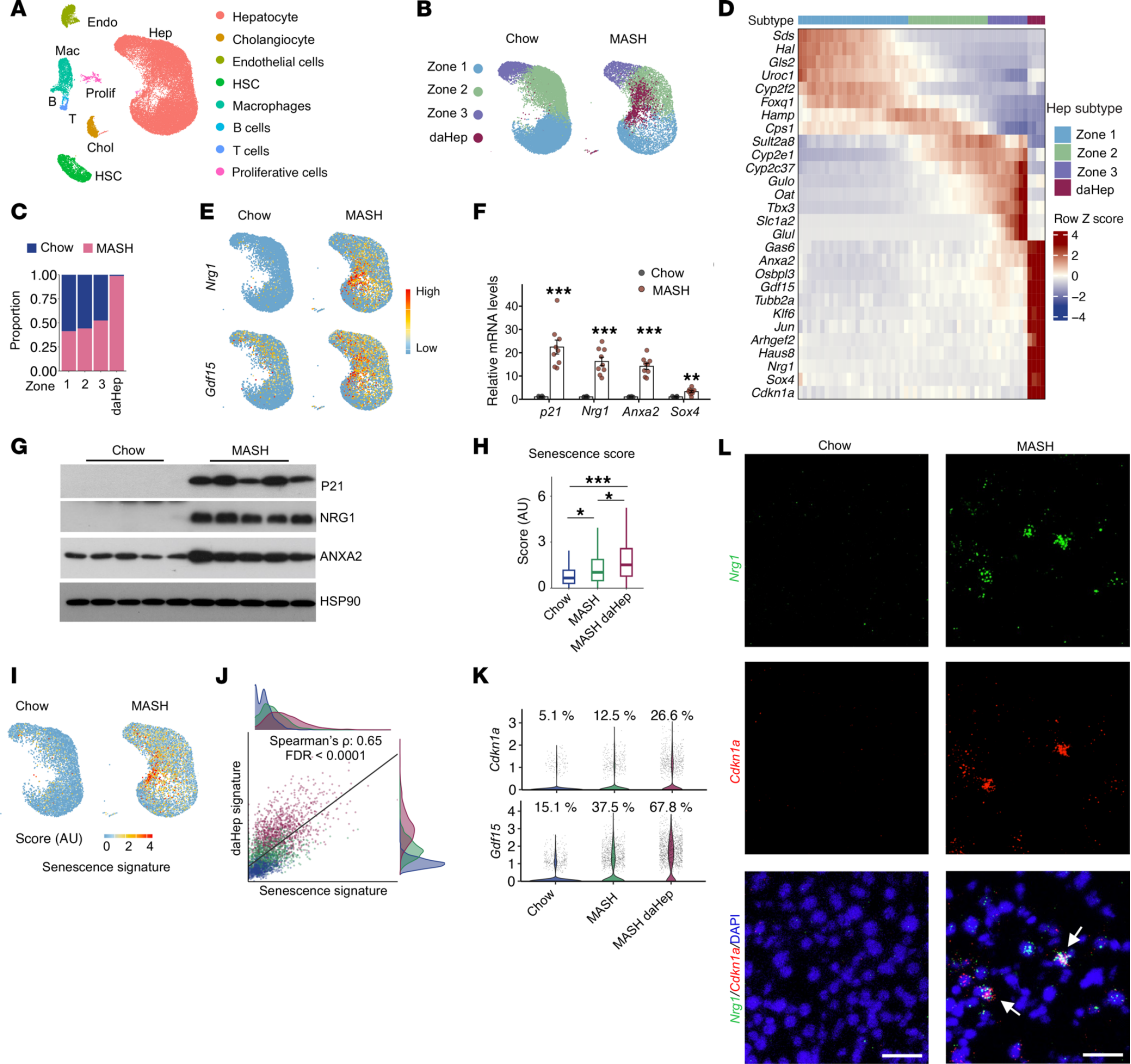
Single-nucleus RNA-seq analysis of healthy and diet-induced MASH livers. (**A**) UMAP clustering analysis of snRNA-seq dataset of 36,284 liver nuclei. (**B**) Hepatocyte subclusters in chow and MASH livers. (**C**) Relative proportion of chow and MASH hepatocytes for each subtype. (**D**) Heatmap illustrating the expression of marker genes for different hepatocyte subtypes. Hepatocytes in zones 1–3 were sorted along their coordinates in the first principal component (PC1) dimension. Each column represents average values binned from 500 neighboring cells. (**E**) Feature plots showing *Nrg1* and *Gdf15* expression in hepatocytes. (**F**) qPCR analysis of hepatic gene expression (chow, *n* = 4; MASH, *n* = 9). Statistical comparisons were conducted using 2-tailed unpaired Student’s *t* test. **P < 0.01, ***P < 0.001. (**G**) Immunoblotting analysis of total liver lysates. (**H**) Senescence gene score for different hepatocyte subtypes. Significance was estimated via 100 subsampling permutations (*n* = 30) using Wilcoxon’s rank-sum tests. Resulting *P* values were adjusted for multiple testing using the Benjamini-Hochberg FDR procedure. *P < 0.05, ***P < 0.001. (**I**) Visualization of hepatocyte senescence scores on UMAP coordinates. (**J**) Correlation between daHep and senescence gene signatures. (**K**) Violin plots of the normalized expression of *Cdkn1a* and *Gdf15* in different hepatocyte subtypes. (**L**) RNA scope analysis of *Nrg1* and *Cdkn1a* expression in chow and MASH liver sections. Arrows indicate colocalization. Scale bars: 50 μm.

Hepatocytes and NPCs in the liver undergo cell type–specific perturbations in gene expression and cellular functions during MASH. To delineate the nature of disease-associated transition of cell states and remodeling of liver microenvironment, we performed single-nucleus RNA-seq (snRNA-seq) analysis on the livers from mice fed either chow or a high-fat high-fructose MASH diet for 5 months (14). Uniform manifold approximation and projection (UMAP) clustering of transcriptomic data revealed 8 major cell types in the liver, including hepatocytes, cholangiocytes, endothelial cells, HSCs, macrophages, B cells, T cells, and proliferating cells (Figure 1A and Supplemental Figure 1, A and B; supplemental material available online with this article; https://doi.org/10.1172/JCI199303DS1). Further clustering within the hepatocyte population revealed robust zonation (Figure 1, B–D, and Supplemental Figure 1C), a hallmark of spatial heterogeneity of liver parenchymal cells (12). Hepatocytes exhibited gradient expression of zonal markers (e.g., *Hal, Hamp,* and *Glul*), defining periportal (zone 1), midlobular (zone 2), and pericentral (zone 3) subtypes. Beyond the subclusters corresponding to liver zonation, we identified a hepatocyte subpopulation with a unique transcriptomic signature that was rare in healthy liver but markedly expanded upon diet-induced MASH (Figure 1, B and C). These deHep shared the transcriptomic features of midlobular and pericentral hepatocytes, while displaying highly enriched expression of a panel of genes, including *Nrg1* (neuregulin 1), *Anxa2* (annexin 2), *Gdf15*, and transcription factors *Sox4*, *Klf6*, and *Jun* (Figure 1, D and E, and Supplemental Figure 1D). Quantitative PCR (qPCR) and immunoblotting analyses confirmed that, compared with mice fed a control chow diet, hepatic mRNA and protein levels of Nrg1 and Anxa2 were markedly increased upon diet-induced MASH (Figure 1, F and G). We performed immunofluorescence staining using cytokeratin 8 (CK8), a pan-hepatocyte marker, and ANXA2. While hepatocytes expressing ANXA2 were nearly absent in chow liver, ANXA2^+^ hepatocytes were readily observed in midlobular and pericentral zones in MASH liver (Supplemental Figure 1E). ANXA2^+^ hepatocytes were recently identified in the settings of acute liver failure in humans and in mouse models of chronic liver disease and liver cancer (23, 39). This hepatocyte subpopulation exhibits a migratory phenotype and serves an important role in wound closure following liver injury.

Recent work has demonstrated that daHep harbor increased DNA copy number variations and may contribute to the development of liver malignancy (23). Despite this, the molecular characteristics and significance of daHep in MASH progression remain largely unknown. We performed differential gene expression to identify genes enriched in daHep compared with non-daHep in MASH liver. Pathway analyses indicated that daHep exhibited reduced expression of genes associated with core hepatic metabolic functions, such as cholesterol and bile acid biosynthesis, protein synthesis, amino acid and xenobiotic metabolism, and urea cycle (Supplemental Figure 1F). Gene set enrichment analyses revealed that daHep-enriched genes were strongly associated with cellular senescence and activation of p53 pathway (Supplemental Figure 2A). Recent studies have implicated cellular senescence as an emerging pathogenic factor in metabolic liver disease progression (25, 27). To explore whether different hepatocyte subpopulations exhibit varying degree of senescence, we compiled gene lists associated with cellular senescence (40) and computed senescence scores for individual hepatocytes. Compared with healthy and non-daHep MASH hepatocytes, daHep from MASH livers exhibited the highest senescence score (Figure 1, H and I). Furthermore, we observed a highly significant correlation between daHep and senescence gene signatures within the daHep subpopulation (Figure 1J). Accordingly, the percentage of hepatocytes expressing *Cdkn1a* (encoding p21) and *Gdf15*, 2 genes enriched in senescent cells, was higher in the daHep subpopulation, compared with healthy and non-daHep MASH hepatocytes (Figure 1K). Consistently, RNAscope in situ hybridization revealed that both Nrg1^+^ and Cdkn1a^+^ cells were markedly induced in the liver upon diet-induced MASH, and importantly, a subset of Nrg1^+^ hepatocytes displayed strong coexpression of *Cdkn1a,* as shown by dual-color RNAscope analysis (Figure 1L). Gene expression analysis indicated that hepatic p21 mRNA and protein levels were substantially increased upon diet-induced MASH (Figure 1, F and G). Immunofluorescence staining also revealed that, while P21^+^ cells were rare in healthy liver, they could be readily detected in MASH liver (Supplemental Figure 2B). Furthermore, we observed the induction of SA-β-GAL–positive cells in the liver and increased hepatic expression of genes linked to senescence (*Cdkn1a, Jun*) and SASP, including *Gdf15*, *Tgfb1*, *Mmp12*, *Lcn2*, and *Lgals3*, upon diet-induced MASH (Supplemental Figure 2, B–D). Together, these results illustrate cellular senescence as a prominent feature of daHep during MASH pathogenesis.

### THEMIS induction is a core feature of transcriptomic remodeling within hepatocytes during MASH.

**Figure 2:**
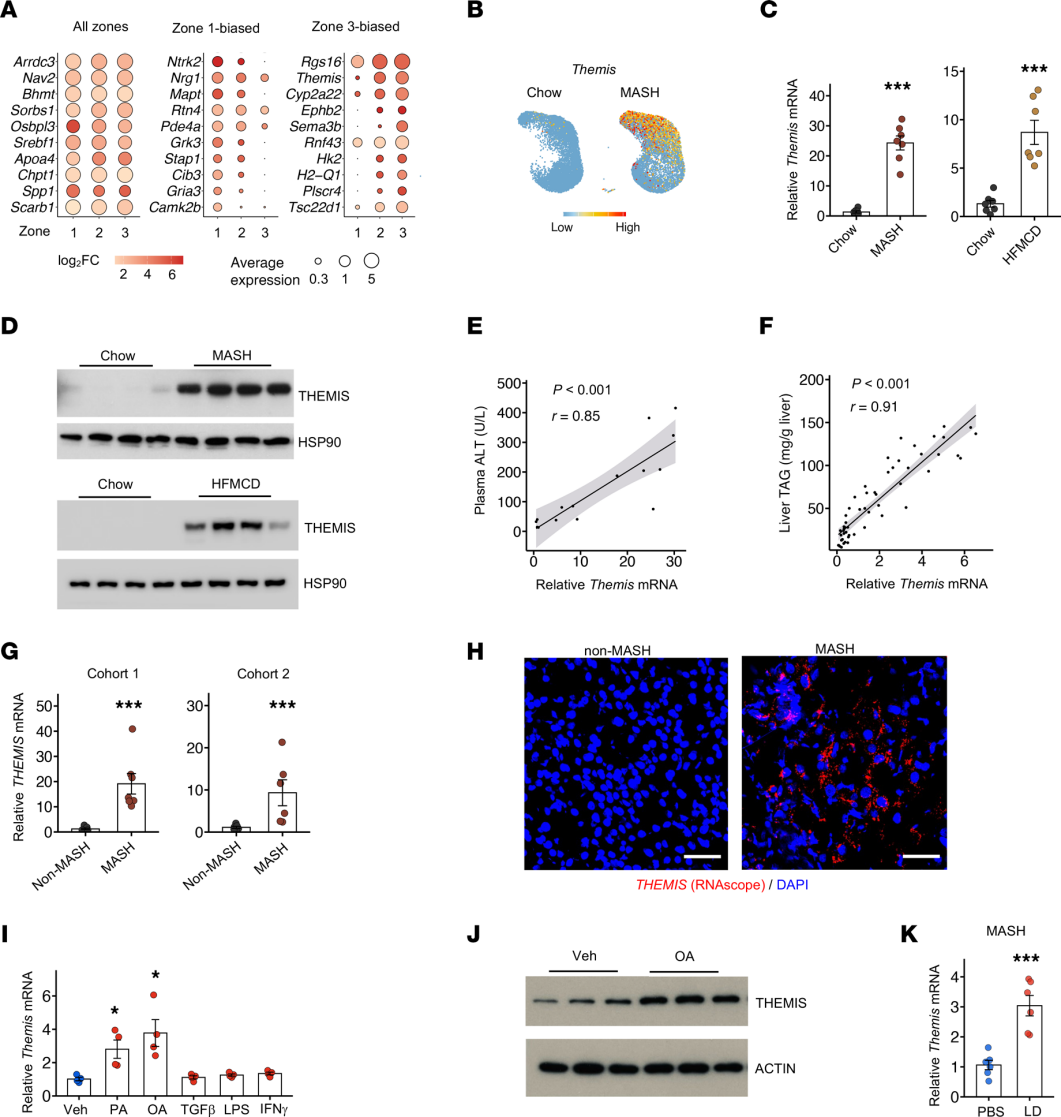
Induction of Themis expression in mouse and human MASH livers. (**A**) Classification of top differentially expressed genes according to their zonal expression patterns. (**B**) Feature plots showing *Themis* expression in chow and MASH hepatocytes. (**C**) qPCR analysis of *Themis* expression in control and diet-induced MASH livers (*n* = 7 per group). (**D**) Immunoblotting analysis of total liver lysates. (**E**) Correlation analysis of hepatic *Themis* expression with plasma ALT levels in a cohort of MASH diet–fed mice (*n* = 15). (**F**) Correlation analysis of hepatic *Themis* expression with liver TAG content in a cohort of diet-induced obese mice (*n* = 55). (**G**) qPCR analysis of hepatic *THEMIS* expression in 2 cohorts of individuals without MASH (cohort 1: *n* = 8, cohort 2: *n* = 6) and individuals with MASH (cohort 1: *n* = 7, cohort 2: *n* = 6). (**H**) RNA scope analysis of *THEMIS* expression in non-MASH and MASH human liver biopsies. (**I**) qPCR analysis of *Themis* expression in primary hepatocytes treated with vehicle, 300 μM palmitic acid (PA), 300 μM oleic acid (OA), 5 ng/mL TGF-β, 20 ng/mL LPS, and 20 ng/mL IFN-γ for 24 hours. (**J**) Immunoblotting analysis of total lysates from treated hepatocytes. (**K**) qPCR analysis of hepatic *Themis* expression in MASH diet–fed mice receiving either PBS or lipid droplet treatment via tail vein injection (*n* = 6). Data in **C**, **G**, **I**, and **K** represent mean ± SEM; statistical comparisons were conducted using 2-tailed unpaired Student’s *t* test for **C**, **G**, and **K**) and with 1-way ANOVA with post hoc Tukey’s HSD test in **I**. *P < 0.05, ***P < 0.001. Scale bars: 50 μm (**H**).

The mechanisms that govern hepatocyte senescence and daHep induction and expansion in the MASH liver remain unknown. To explore this, we performed differential gene expression analysis on hepatocytes, stratified by their zonal locations. This analysis identified genes exhibiting differential expression between chow and MASH hepatocytes in all 3 zones and those displaying zone-specific patterns (Figure 2A). Notably, mRNA expression of *Themis*, a regulator of T cell receptor signaling thymocyte development (41–43), was nearly absent in healthy hepatocytes and exhibited a marked increase in midlobular and pericentral hepatocytes in MASH liver (Figure 2, A and B). In contrast, THEMIS mRNA expression in liver T cells remained comparable between chow and MASH groups in our single-cell and snRNA-seq datasets (Supplemental Figure 3, A and B). While the role of THEMIS in T cell receptor signaling and lineage development has been well-characterized (44, 45), whether this factor modulates hepatocyte signaling and liver disease progression have remained unexplored. We confirmed markedly increased mRNA and protein expression of THEMIS in the liver from two diet-induced MASH models (Figure 2, C and D). Hepatic *Themis* mRNA expression was strongly associated with plasma alanine aminotransferase (ALT) levels, a marker of liver injury, in a cohort of MASH mice exhibiting varying degrees of liver injury (Figure 2E). Additionally, *Themis* mRNA expression displayed a robust correlation with liver triglyceride (TAG) content in mice fed a high-fat diet (Figure 2F). Importantly, hepatic expression of *THEMIS* was significantly induced in human MASH livers compared with non-MASH controls, as revealed by qPCR and RNAscope analyses (Figure 2, G and H). Elevated *THEMIS* expression was consistently observed in several human liver bulk RNA-seq datasets from patients with MASH, compared with that in non-MASH or MASLD cohorts (Supplemental Figure 3C). These results demonstrate that THEMIS induction in liver is a conserved feature of human and murine MASH.

We postulated that hepatic *Themis* expression may respond to pathophysiological cues within the MASH liver microenvironment. To test this, we exposed cultured primary hepatocytes to a panel of known metabolic stressors (fatty acids) and cytokines associated with MASH and examined their effects on *Themis* gene expression. While TGF-β, TNF-α, and IFN-γ elicited minimal effects on *Themis* mRNA expression, both palmitic acid and oleic acids substantially increased *Themis* mRNA and protein levels in primary hepatocytes (Figure 2, I and J). We recently demonstrated that LD exposed by injured hepatocytes serve as a danger signal that exacerbates MASH pathologies (21). Interestingly, treatment of diet-induced MASH mice via tail vein injection of isolated LD significantly increased *Themis* mRNA levels in the liver (Figure 2K). These findings suggest that increased hepatic lipid flux may serve as a driver of THEMIS induction during metabolic liver disease pathogenesis.

### THEMIS deficiency exacerbates hepatocyte senescence and diet-induced MASH progression.

**Figure 3:**
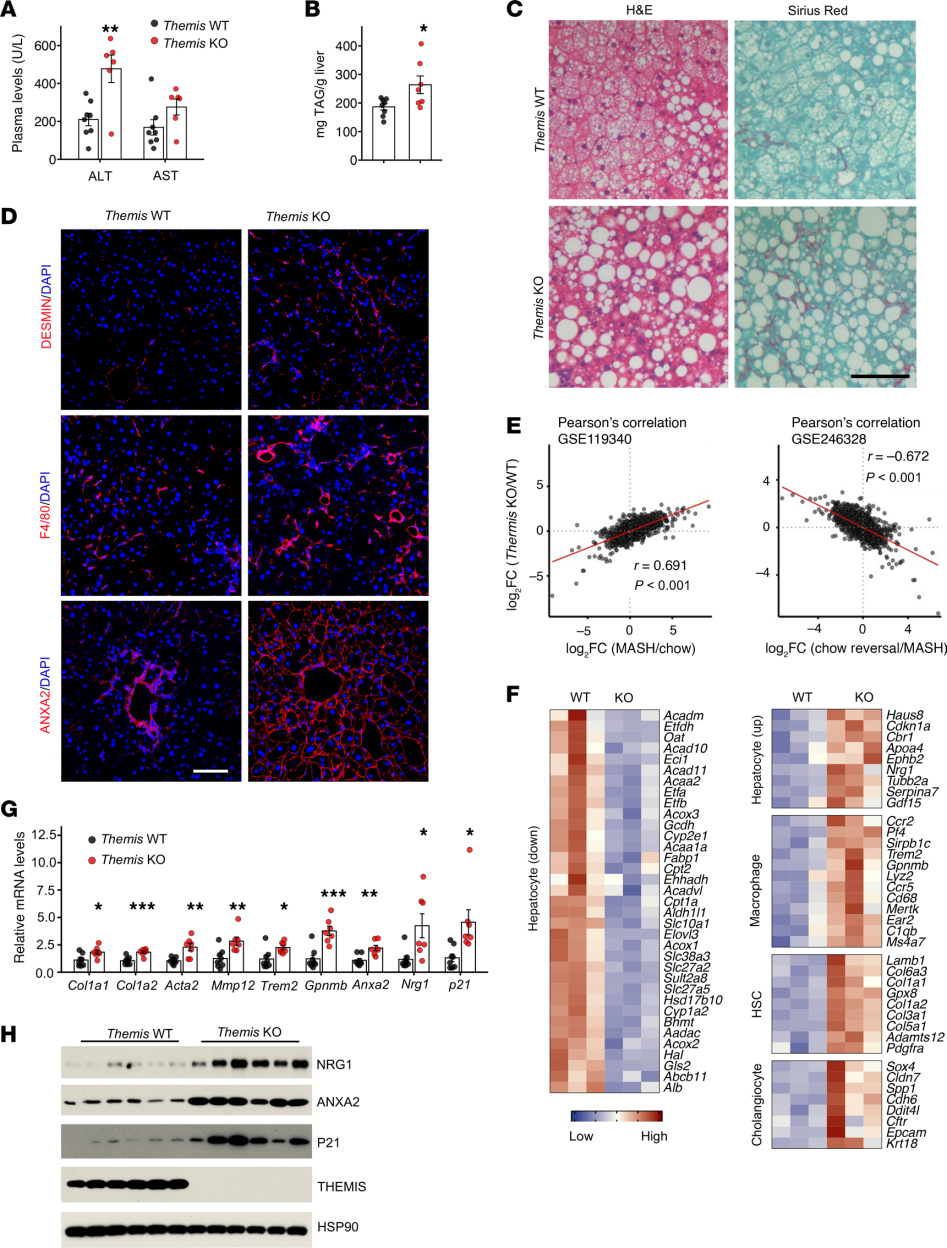
*Themis* deficiency accelerates diet-induced MASH progression. WT (*n* = 8) and *Themis*-KO (*n* = 7) male mice were fed MASH diet for 12 weeks. (**A**) Plasma ALT and AST levels. (**B**) Liver TAG content. (**C**) H&E and Sirius red staining of liver sections. (**D**) Immunofluorescence staining of liver sections. (**E**) Correlation analysis of hepatic gene expression between Themis inactivation and diet-induced MASH (left) or MASH resolution (right). (**F**) Heatmaps illustrating the effects of THEMIS ablation on the expression of genes enriched in different liver cell types. (**G**) qPCR analysis of hepatic gene expression in WT (*n* = 8) and Themis-KO (*n* = 7) livers. (**H**) Immunoblotting of total liver lysates. Data in **A**, **B**, and **G** represent mean ± SEM; 2-tailed unpaired Student’s *t* test. *P < 0.05, **P < 0.01, ***P < 0.001. Scale bars: 100 μm (**C** and **D**).

In response to pathophysiological stressors, hepatocytes engage diverse signaling pathways that mediate adaptive stress response and promote the restoration of homeostasis. In contrast, maladaptive responses may exacerbate metabolic dysfunctions and liver injury, thereby accelerating disease progression. To elucidate whether THEMIS plays a hepatoprotective or pathogenic role in metabolic liver disease, we examined the effect of THEMIS deletion on the development of diet-induced MASH pathologies in mice. Under chow-fed conditions, WT and *Themis*-KO littermates exhibited comparable plasma ALT and aspartate aminotransferase (AST) levels, metabolic parameters, and liver histology (Supplemental Figure 4). Following 3 months of MASH diet feeding, however, *Themis*-KO mice displayed significantly higher plasma ALT levels and developed more severe hepatic steatosis, as evidenced by elevated liver weight and TAG content and H&E histology (Figure 3, A–C, and Supplemental Figure 5, A and B). Plasma nonesterified fatty acids and TAG levels remained comparable between the 2 groups (Supplemental Figure 5C). Sirius red staining revealed more extensive pericellular fibrosis in *Themis*-KO livers, which was accompanied by increased infiltration of F4/80^+^ macrophages and induction of DESMIN^+^ HSCs (Figure 3, C and D, and Supplemental Figure 5D).

We performed bulk RNA-seq analysis on 3 pairs of WT and *Themis*-KO livers to examine the effects of THEMIS on global gene expression. Differential expression analysis revealed 485 upregulated and 295 downregulated genes (2-fold cutoff) associated with THEMIS inactivation (Supplemental Figure 5E). The transcriptomic profile induced by THEMIS deficiency closely resembled that observed in diet-induced MASH, while exhibiting an inverse correlation with gene expression changes linked to dietary switch–mediated MASH resolution (Figure 3E). Among the differentially expressed genes, many hepatocyte-enriched genes involved in lipid metabolism and β-oxidation were downregulated, whereas NPC-enriched genes related to cell adhesion, migration, extracellular matrix components, and immune response were upregulated in the *Themis*-KO group (Figure 3F). Cell-type deconvolution analysis from the bulk RNA-seq data revealed increased proportion of cholangiocytes, macrophages, and HSCs (Supplemental Figure 5F). qPCR analysis confirmed significant upregulation of genes implicated in liver fibrosis (*Col1a1*, *Col1a2*, *Acta2*), inflammation (*Trem2*, *Gpnmb*, *Mmp12*), and daHep induction (*Axna2*, *Nrg1*, *Cdkn1a*) in the *Themis*-KO group compared with the control groups (Figure 3G and Supplemental Figure 5G). Consistent with the transcriptomic data, protein levels of NRG1, ANXA2, and P21 were markedly elevated, alongside increased abundance of ANXA2^+^ hepatocytes in THEMIS-deficient livers (Figure 3, D and H). Collectively, these findings strongly support a hepatoprotective role for MASH-associated THEMIS induction in mitigating the progression of metabolic liver disease.

### Hepatocyte-specific ablation of THEMIS worsens diet-induced MASH pathologies.

**Figure 4:**
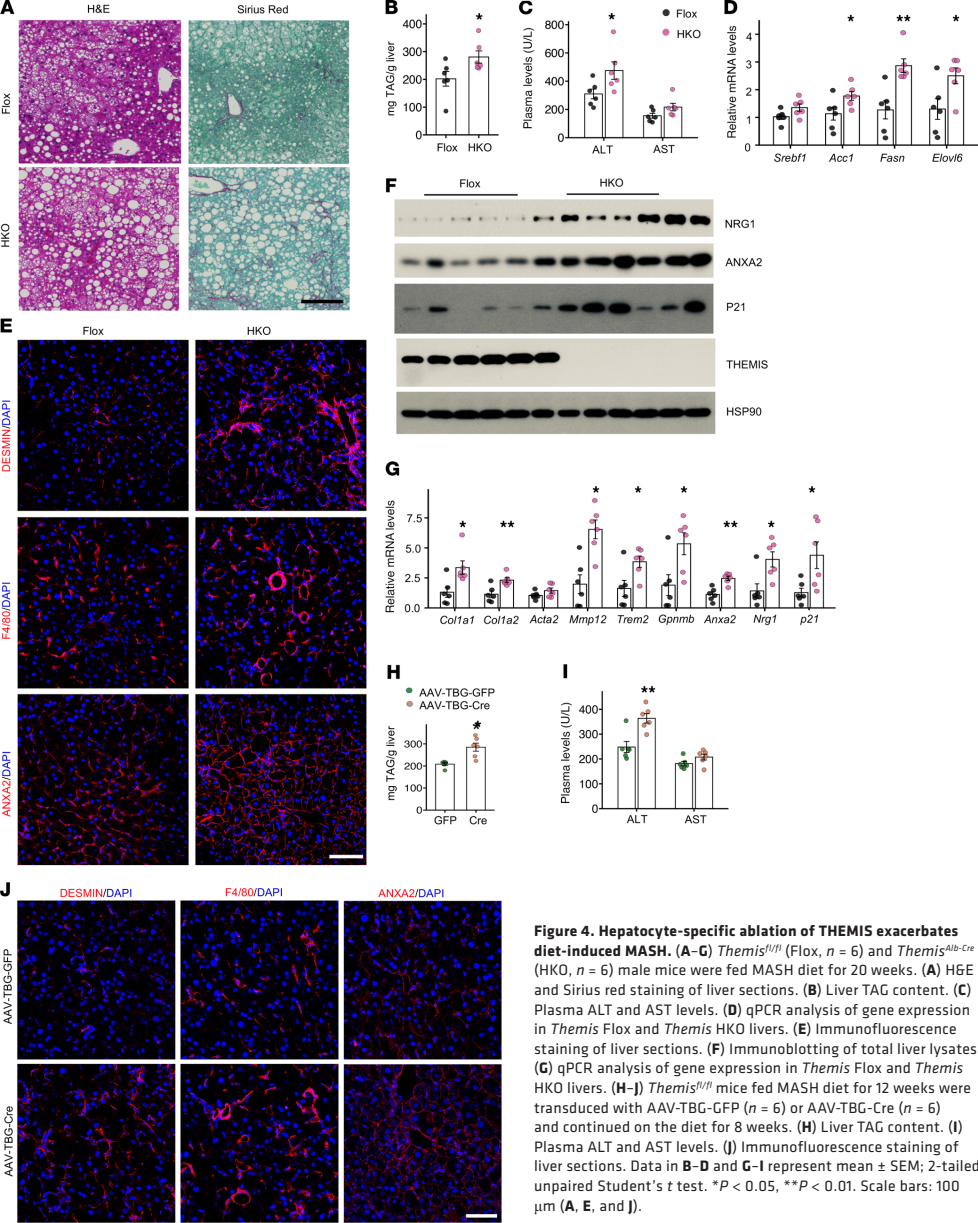
Hepatocyte-specific ablation of THEMIS exacerbates diet-induced MASH. (**A**–**G**) *Themis^fl/fl^* (Flox, *n* = 6) and *Themis^Alb-Cre^* (HKO, *n* = 6) male mice were fed MASH diet for 20 weeks. (**A**) H&E and Sirius red staining of liver sections. (**B**) Liver TAG content. (**C**) Plasma ALT and AST levels. (**D**) qPCR analysis of gene expression in *Themis* Flox and *Themis* HKO livers. (**E**) Immunofluorescence staining of liver sections. (**F**) Immunoblotting of total liver lysates. (**G**) qPCR analysis of gene expression in *Themis* Flox and *Themis* HKO livers. (**H**–**J**) *Themis^fl/fl^* mice fed MASH diet for 12 weeks were transduced with AAV-TBG-GFP (*n* = 6) or AAV-TBG-Cre (*n* = 6) and continued on the diet for 8 weeks. (**H**) Liver TAG content. (**I**) Plasma ALT and AST levels. (**J**) Immunofluorescence staining of liver sections. Data in **B**–**D** and **G**–**I** represent mean ± SEM; 2-tailed unpaired Student’s *t* test. *P < 0.05, **P < 0.01. Scale bars: 100 μm (**A**, **E**, and **J**).

Given the abundant expression of THEMIS in T lymphocytes, it is plausible that T cell dysfunction may contribute to the exacerbation of MASH phenotypes observed in whole-body *Themis*-KO mice. To delineate the hepatocyte-intrinsic role of THEMIS in MASH pathophysiology, we generated hepatocyte-specific *Themis*-knockout (HKO) mice by crossing *Themis*^fl/fl^ (Flox) animals with Albumin-Cre–transgenic (Alb-Cre–transgenic) mice. Under standard chow diet conditions, Flox and HKO mice exhibited comparable physiological and metabolic parameters, including body weight, tissue mass, fasting blood glucose, and plasma ALT and AST levels as well as hepatic gene expression profiles (Supplemental Figure 6). In contrast, HKO mice exhibited higher liver weight, aggravated hepatic steatosis and liver injury following MASH diet feeding, compared with Flox controls (Figure 4, A–C, and Supplemental Figure 7, A–D). Consistently, qPCR analysis revealed significant upregulation of genes encoding key enzymes involved in de novo lipogenesis (DNL), including *Acc1, Fasn* and *Elovl6*, in the HKO livers (Figure 4D). Sirius red staining revealed marked increased hepatic fibrosis in HKO livers, concomitant with elevated numbers of DESMIN^+^ HSCs, and the formation of crown-like structures surrounded by F4/80^+^ macrophages (Figure 4, A and E). This was further supported by markedly higher expression of genes associated with liver fibrosis and inflammatory responses in the HKO livers than the control group (Figure 4G and Supplemental Figure 7E). Compared with that in controls, mRNA and protein expression of *Nrg1*, *Anxa2*, and *Cdkn1a* (p21) was significantly upregulated in the HKO livers (Figure 4, F and G), consistent with more abundant ANXA2^+^ hepatocytes (Figure 4E). In contrast, mRNA expression of T cell exhaustion markers, including *Havcr2*, *Lag3* and *Pdcd1*, was not significantly altered in HKO livers (Supplemental Figure 7F).

To further interrogate the acute consequences of hepatocyte-specific THEMIS depletion, we administered recombinant adeno-associated virus (AAV) vectors encoding either GFP or Cre recombinase driven by *TBG* promoter into MASH diet–fed *Themis* Flox mice. Consistent with the phenotype observed in the HKO model, acute ablation of *Themis* in hepatocytes led to increased hepatic TAG accumulation, exacerbated liver injury and fibrosis, augmented macrophage infiltration, and more ANXA2^+^ hepatocytes (Figure 4, H–J, and Supplemental Figure 7, G–K). Collectively, these findings demonstrate that hepatocyte-intrinsic THEMIS exerts a cell-autonomous protective role in mitigating diet-induced MASH progression.

### Hepatic THEMIS shapes liver cell transcriptomic states during MASH.

**Figure 5:**
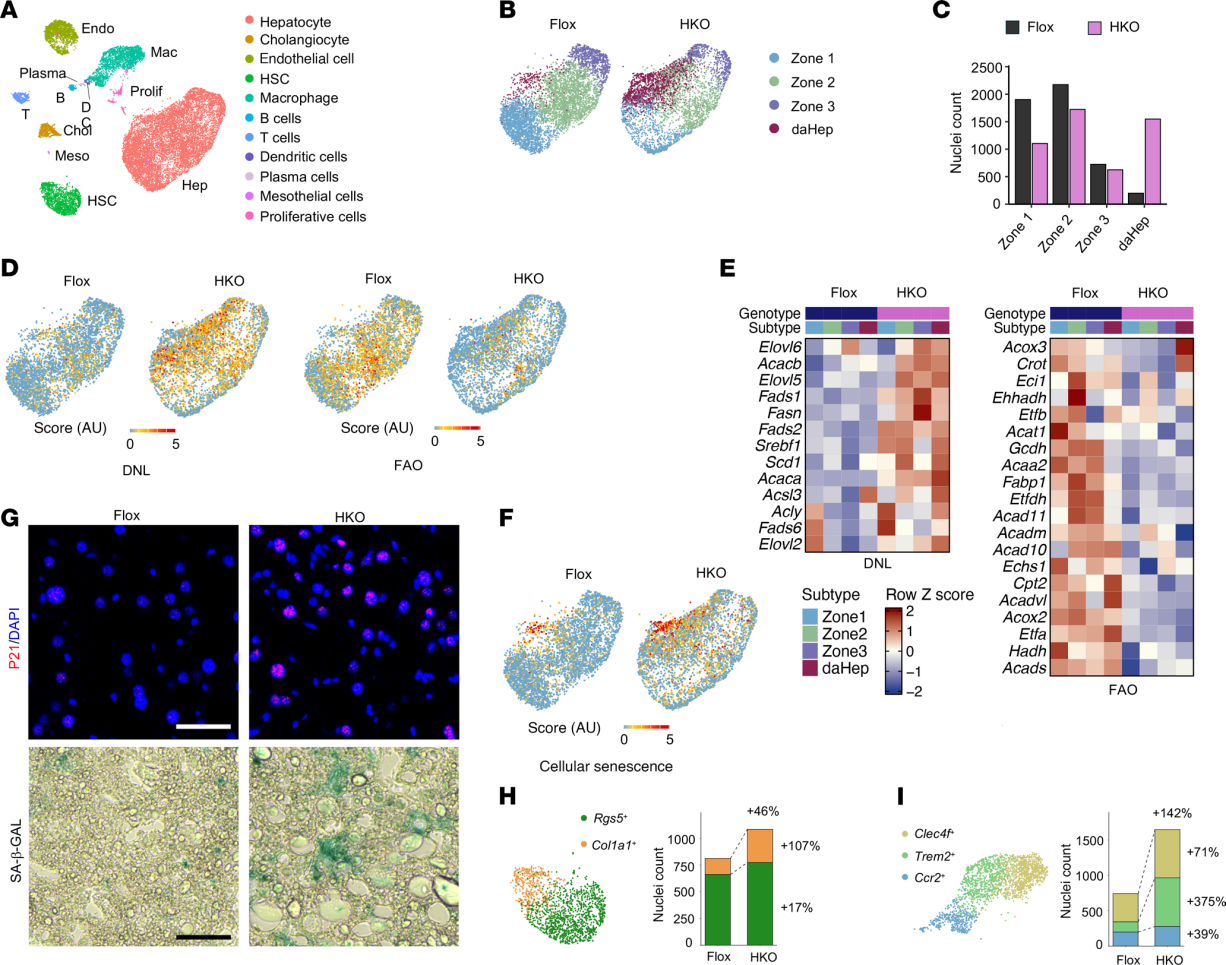
THEMIS inactivation reprograms hepatocyte and nonparenchymal cell transcriptomes in MASH liver. (**A**) UMAP plot following clustering analysis of single nuclei from *Themis^fl/fl^* (Flox) and *Themis^Alb-Cre^* (HKO) mouse livers. (**B**) UMAP plots of subclustered hepatocyte subtypes. (**C**) Cell count for each hepatic subtype in Flox and HKO livers. (**D**) UMAP plots of DNL and FAO gene activity scores for individual hepatocytes. (**E**) Heatmaps depicting DNL and FAO gene expression in Flox and HKO hepatocytes. (**F**) UMAP plots of senescence gene activity score for hepatocytes. (**G**) Immunofluorescence staining and senescence-associated β-galactosidase (SA-β-GAL) staining of liver sections. (**H**) Subclustering analysis of HSCs. (**I**) Subclustering analysis of liver macrophages. Scale bars: 50 μm (**G**).

Central to MASH pathogenesis is the pervasive transcriptomic reprogramming and dynamic cell state transitions in both hepatocyte and NPC populations. To delineate the role of hepatic THEMIS in shaping liver cell heterogeneity in MASH, we conducted snRNA-seq analysis on liver nuclei isolated from 4 pairs of MASH diet–fed *Themis* Flox and HKO mice. As expected, we recovered all major liver cell types for both genotypes (Figure 5A and Supplemental Figure 8, A and B). Within the hepatocyte compartment, UMAP analysis identified 4 discrete subpopulations corresponding to distinct liver zones and daHep (Figure 5B). Cell count analysis revealed a marked expansion of daHep in *Themis* HKO livers (Figure 5C), underscoring the critical role of THEMIS in restraining daHep induction during MASH. As shown above, both whole-body and hepatocyte-specific THEMIS inactivation aggravated hepatic steatosis, likely a consequence of underlying disruptions of hepatic lipid metabolism. To further dissect these effects, we computed pathway activity scores for DNL and fatty acid β-oxidation (FAO) based on curated gene sets and projected these scores onto the UMAP representation of hepatocytes. *Themis* HKO hepatocytes exhibited elevated DNL scores and increased lipogenic gene expression relative to controls, whereas FAO scores and the expression of FAO genes were reduced (Figure 5, D and E). Assessment of cellular senescence signatures showed that *Themis* HKO livers contained a greater proportion of hepatocytes expressing senescence-associated gene profiles, with these cells largely overlapping the daHep cluster (Figure 5F). Furthermore, *Themis* HKO livers exhibited a pronounced increase in P21-positive nuclei and SA-β-GAL staining, indicative of more prevalent hepatocyte senescence as a consequence of THEMIS inactivation (Figure 5G). In contrast, *Themis* deficiency did not affect hepatocyte proliferation, as revealed by comparable proliferation scores and mRNA expression of *Pcna*, a marker of cell proliferation, in the hepatocyte cluster between the 2 genotypes (Supplemental Figure 8, C and D).

We next investigated how hepatocyte-specific ablation of THEMIS affects the NPC compartment of the liver microenvironment during diet-induced MASH. Previous studies have identified HSC subpopulations corresponding to quiescent and activated phenotypes in MASH livers (17, 19). Consistent with these reports, UMAP clustering revealed 2 distinct HSC populations: *Rgs5*^+^ quiescent HSCs and *Col1a1*^+^ activated HSCs (Figure 5H). Cell count analysis demonstrated that, compared with that of controls, total HSC numbers increased by approximately 46% in HKO group, with the *Col1a1^+^* activated HSC subpopulation exhibiting a striking 107% expansion (Figure 5H and Supplemental Figure 8E). Macrophage heterogeneity is a well-established feature of MASH, and 3 principal macrophage subtypes were identified: resident Kupffer cells, monocyte-derived macrophages, and *Trem2*^+^ macrophages (14, 46). The overall macrophage abundance showed a marked increase in HKO livers relative to controls, with the *Trem2*^+^ macrophage subset displaying the most pronounced expansion by nearly 4 folds following hepatocyte THEMIS ablation (Figure 5I and Supplemental Figure 8F). These findings underscore a pivotal role for hepatocyte-intrinsic THEMIS signaling in modulating both parenchymal and nonparenchymal compartments, thereby shaping the cellular landscape of the liver microenvironment during MASH progression.

### THEMIS attenuates hepatocyte senescence through restraining ERK activation.

**Figure 6:**
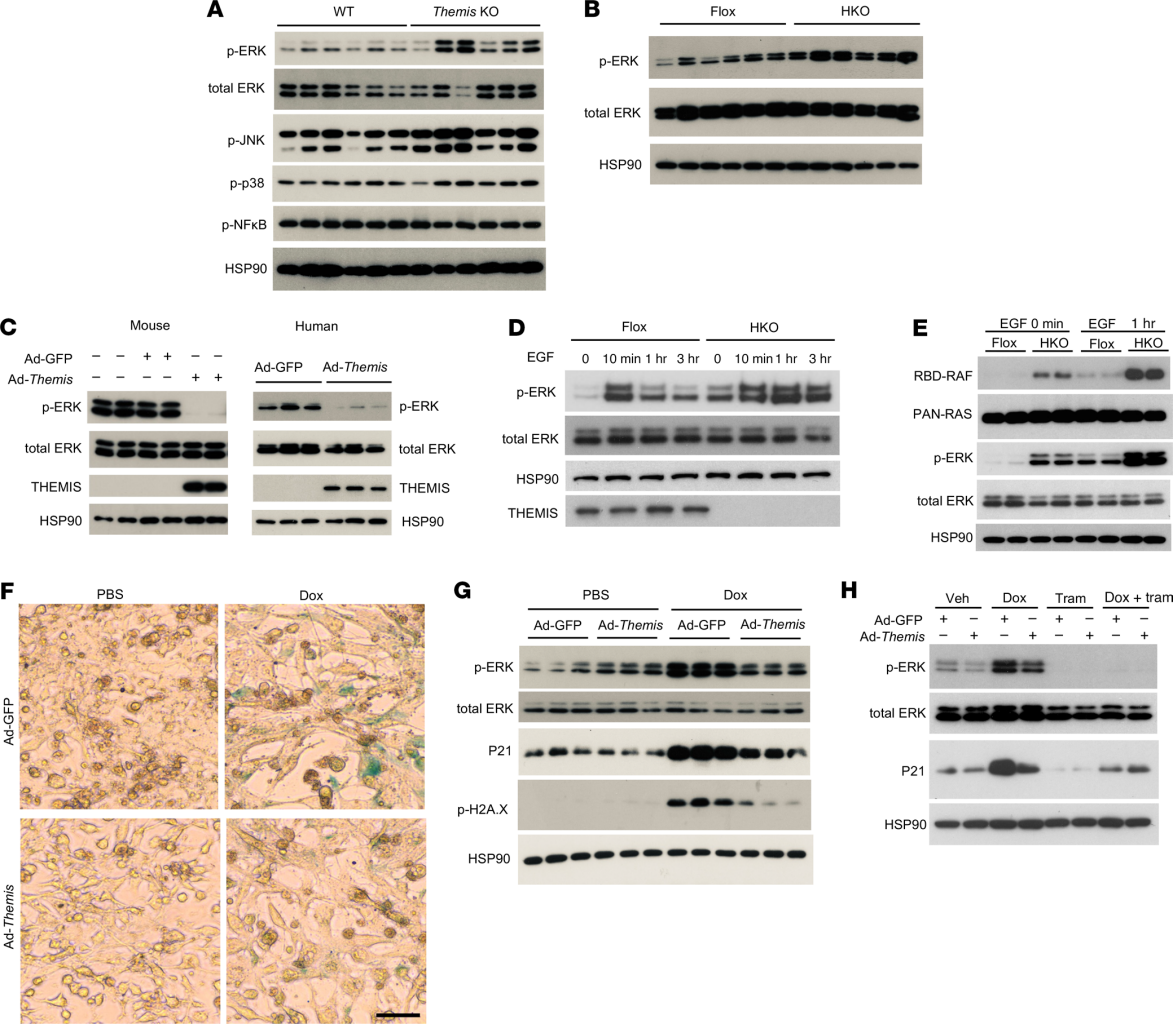
Themis regulates senescence in a cellular autonomous manner via Erk signaling. (**A**) Immunoblotting of total lysates from WT and *Themis*-KO livers. (**B**) Immunoblotting of total lysates from *Themis* Flox and HKO livers. (**C**) Immunoblotting of total lysates from mouse and human primary hepatocytes transduced with Ad-GFP or Ad-Themis. (**D**) Immunoblotting of *Themis* Flox and HKO mice primary hepatocytes stimulated with 100 ng/mL EGF at indicated time points. (**E**) RAS activity assessment of EGF-treated primary hepatocytes isolated from Flox and HKO mice fed MASH diet for 5 months. (**F** and **G**) Hepa1 cells overexpressing either GFP or Themis were treated with 50 nM doxorubicin (Dox) for 3 days. (**F**) SA-β-GAL staining of Hepa1 cells. (**G**) Immunoblotting of total lysates from Hepa1 cells. (**H**) Immunoblotting of total lysates. Hepa1 cells overexpressing either GFP or Themis were treated with 1 μM Dox for 2 hours, followed by 10 nM MEK inhibitor trametinib treatment for 3 days. Scale bars: 50 μm (**F**).

THEMIS regulates T cell receptor signaling, in part through its effects on the tyrosine phosphatase 1 (SHP1) and ERK pathways (47–50). However, its role in hepatocyte signaling remains unexplored. To investigate the mechanisms underlying THEMIS action in hepatocytes, we analyzed several signaling pathways in the livers from MASH diet–fed WT and *Themis* KO mice. While the phosphorylation status of p38 MAPK and NF-κB was similar between the 2 genotypes, JNK phosphorylation was slightly increased and ERK phosphorylation was consistently elevated in *Themis*-KO livers (Figure 6A). Increase in ERK phosphorylation was similarly observed in the *Themis* HKO livers compared with Flox controls (Figure 6B). Interestingly, expression of hepatic genes associated with the transcriptional response downstream of ERK signaling was elevated in the liver from patients with MASH (Supplemental Figure 3D). To determine whether THEMIS directly inhibits ERK signaling in a cell-autonomous manner, we transduced primary mouse and human hepatocytes with a recombinant adenoviral vector expressing control (Ad-GFP) or THEMIS (Ad-Themis) (Supplemental Figure 9A). Compared with both uninfected and Ad-GFP controls, adenovirus-mediated overexpression of THEMIS strongly attenuated ERK phosphorylation in transduced hepatocytes (Figure 6C), indicating that THEMIS inhibits hepatocyte ERK signaling in a cell-autonomous manner. qPCR analysis indicated that mRNA expression of daHep markers (*Nrg1, Anxa2*) was decreased by THEMIS overexpression in mouse hepatocytes (Supplemental Figure 9B).

To further explore the molecular mechanisms underlying the crosstalk between THEMIS and ERK activation, we isolated primary hepatocytes from Flox and HKO mice fed MASH diet for 5 months and performed treatments with EGF. Compared with control, primary hepatocytes from HKO mice exhibited elevated basal ERK phosphorylation and more sustained ERK activation over a period of 3 hours following EGF stimulation (Figure 6D). THEMIS has been previously demonstrated to physically interact with GRB2, a critical adaptor protein for RAS activation, in thymocytes (51). We observed that GRB2 was present in THEMIS protein complexes affinity-purified from MASH mouse livers (Supplemental Figure 9C). We performed RAS activity assay to assess whether THEMIS directly modulates RAS activation. Compared with controls, *Themis*-deficient hepatocytes exhibited elevated basal level and more robust increase of GTP-bound RAS, the active form of RAS, following EGF stimulation (Figure 6E). Interestingly, treatment of transduced hepatocytes with SHP099, a potent SHP2 inhibitor, did not further augment the inhibitory effects of THEMIS on ERK phosphorylation (Supplemental Figure 9D), suggesting that THEMIS and SHP2 may act in a common pathway upstream of RAS/ERK signaling in hepatocytes.

Aberrant ERK activation has been shown to promote senescence of intestinal epithelial cells (52). In cultured hepatoma cells, treatment with doxorubicin (Dox), a chemotherapeutic drug known to induce DNA damage and cellular senescence (53), strongly stimulated ERK phosphorylation, p21 mRNA and protein expression, and SA-β-Gal accumulation (Figure 6, F and G, and Supplemental Figure 9E). Compared with GFP controls, adenovirus-mediated overexpression of THEMIS attenuated Dox-induced ERK phosphorylation, senescence-related gene expression, and SA-β-Gal staining (Figure 6, F and G, and Supplemental Figure 9E). To further investigate the role of ERK activation in hepatocyte senescence, we treated hepatocytes transduced with Ad-GFP or Ad-THEMIS with Dox in the absence or presence of trametinib, a potent and selective inhibitor for MEK1/2 and ERK activation. As expected, trametinib strongly suppressed ERK activation in response to Dox (Figure 6H). While THEMIS alone can attenuate Dox-induced ERK activation and p21 induction, addition of trametinib abrogated the inhibitory effect of THEMIS on p21 induction (Figure 6H), suggesting that THEMIS acts upstream of ERK to attenuate hepatocyte senescence. These findings demonstrate that THEMIS exerts its inhibitory effects on hepatocyte senescence, at least in part, by suppressing ERK activation.

### THEMIS levels confer protection against hepatocyte senescence and diet-induced MASH.

**Figure 7:**
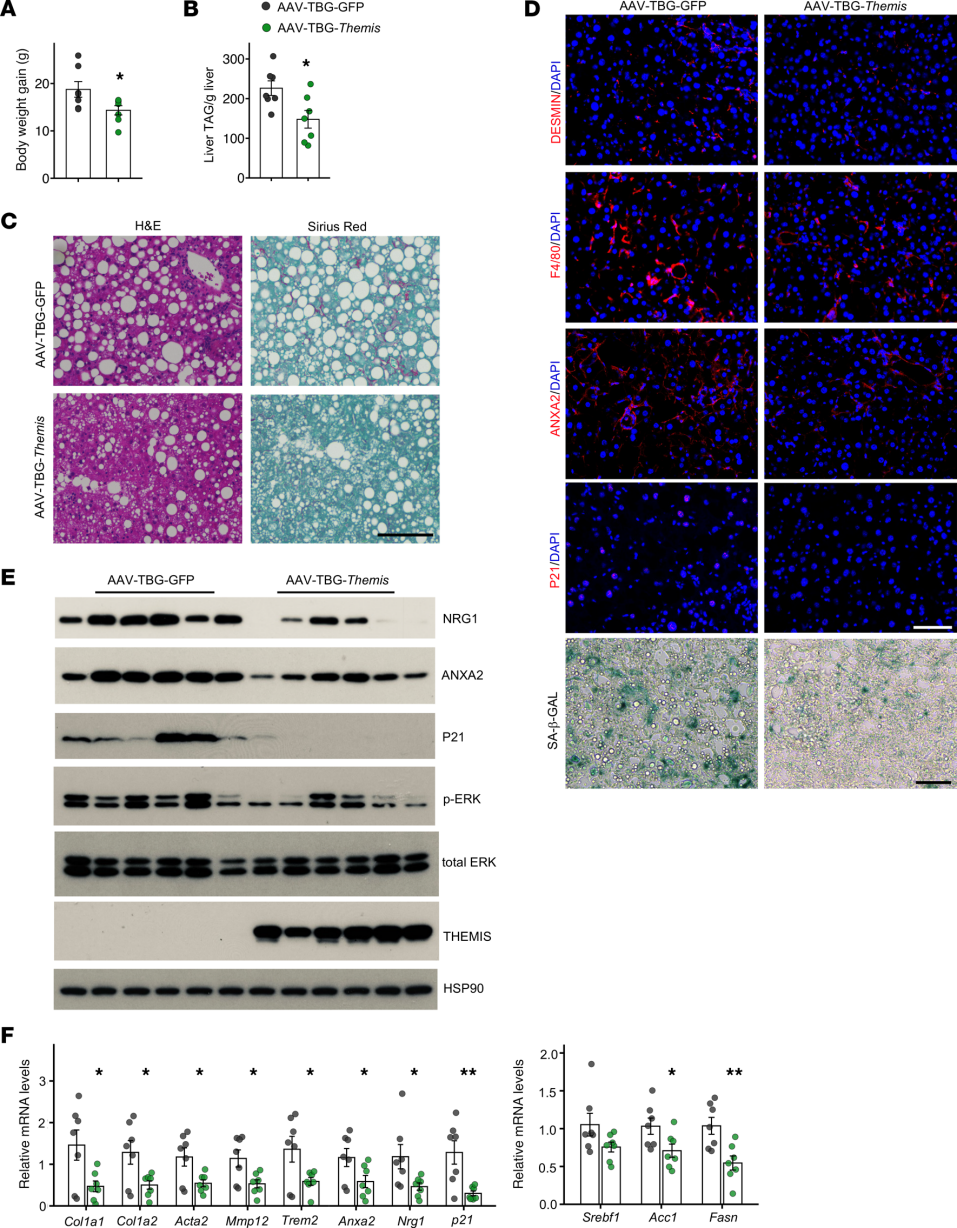
Hepatocyte-specific Themis overexpression attenuates hepatocyte senescence and diet-induced MASH. Male C57BL/6 mice were fed MASH diet for 14 weeks and transduced with either AAV-TBG-GFP (*n* = 7) or AAV-TBG-Themis (*n* = 7) followed by 8 more weeks of feeding. (**A**) Body weight gain. (**B**) Liver TAG measurement. (**C**) H&E and Sirius red staining of liver sections. (**D**) Immunofluorescence and SA-β-GAL staining of liver sections. (**E**) Immunoblotting of total liver lysates. (**F**) qPCR analysis of gene expression. Data in **A**, **B**, and **F** represent mean ± SEM; 2-tailed unpaired Student’s *t* test. *P < 0.05, **P < 0.01. Scale bars: 100 μm (**C** and **D**).

Our findings support a crucial role of THEMIS in orchestrating hepatic adaptive response to metabolic stresses and alleviating metabolic liver disease progression. To determine whether THEMIS is sufficient to confer protection against diet-induced MASH, we constructed a recombinant AAV vector expressing *Themis* under the control of *TBG* promoter, which directs hepatocyte-specific gene expression. We transduced mice fed on MASH diet for 14 weeks with AAV-TBG-GFP or AAV-TBG-THEMIS and continued MASH diet feeding for additional 6 weeks. Mice with AAV-mediated THEMIS overexpression in hepatocytes moderated body weight gain compared with their GFP control counterparts (Figure 7A), along with a trend of decrease in liver weight and liver injury (Supplemental Figure 10, A and B). Histological analysis indicated that AAV-mediated hepatic overexpression of THEMIS attenuated hepatic steatosis, along with lower liver TAG content (Figure 7, B and C). Overexpression of THEMIS improved liver fibrosis, as evidenced by reduced signals from Sirius red staining (Figure 7C) and immunofluorescence staining of DESMIN (Figure 7D). Additionally, AAV-mediated THEMIS overexpression decreased the abundance of F4/80^+^ macrophages in transduced mouse livers (Figure 7D). Analysis of hepatic gene expression indicated that mRNA expression of genes associated with liver fibrosis, TREM2^+^ macrophages, and DNL, but not T cell exhaustion, was significantly decreased by THEMIS overexpression (Figure 7F and Supplemental Figure 10, C and D). Crucially, AAV-mediated overexpression of THEMIS substantially decreased p21 mRNA and protein levels in transduced mouse livers (Figure 7, E and F). P21 immunofluorescence and SA-β-Gal staining were strongly reduced by THEMIS, compared with controls (Figure 7D). This improvement of hepatocyte senescence occurred in parallel with decreased ERK phosphorylation (Figure 7E) and expression of daHep markers, including NRG1 and ANXA2 (Figure 7, D–F). These results illustrate that hepatic overexpression of THEMIS exerts protective effects against diet-induced MASH.

**Figure 8:**
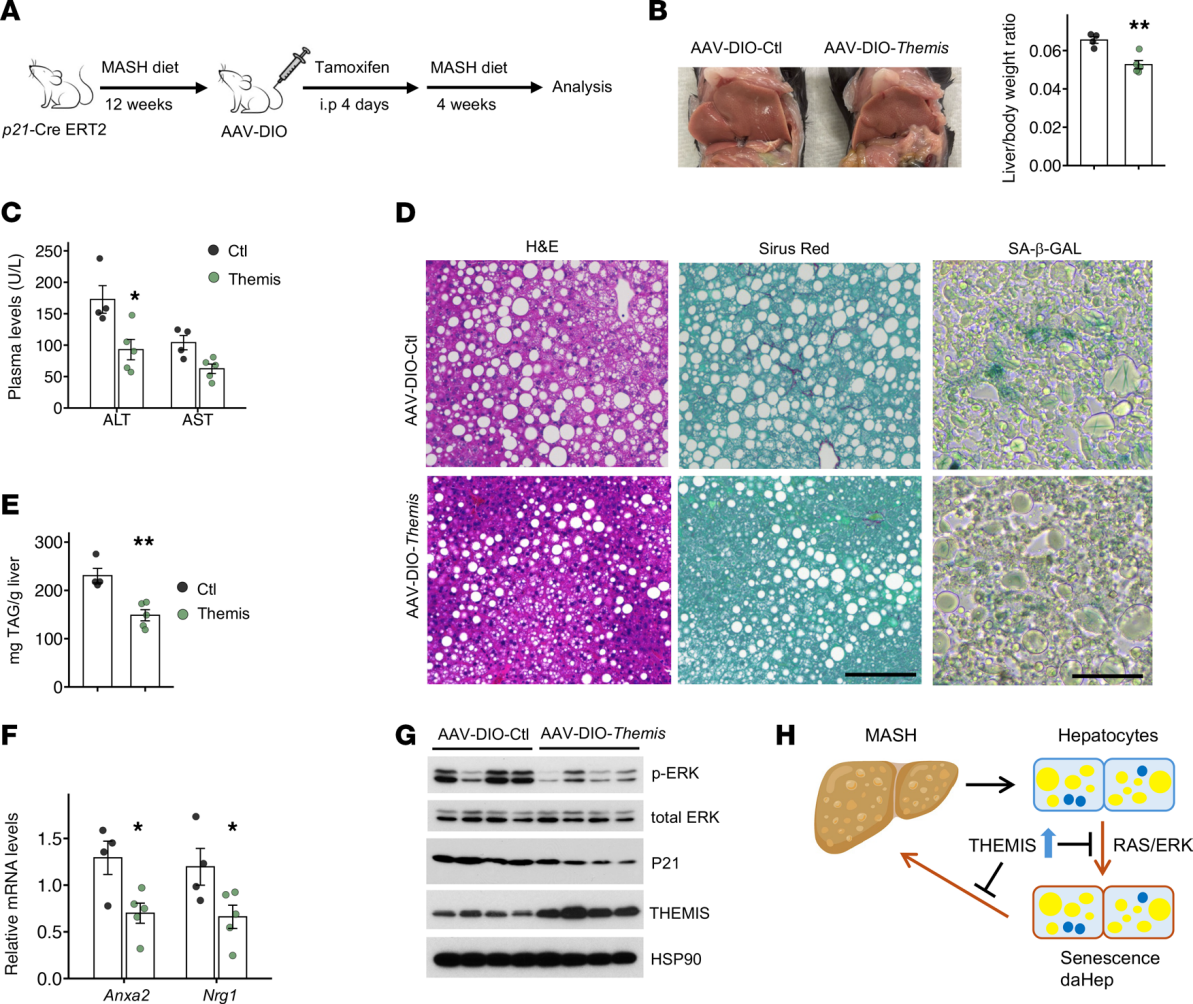
Hepatocyte senescence promotes MASH progression. (**A**) Study design of utilizing *p21*-Cre transgenic mouse strain in combination with AAV-DIO overexpression tool. (**B**) Images of liver and liver/body weight ratio. (**C**) Plasma ALT and AST levels. (**D**) H&E, Sirius red, and SA-β-GAL staining of liver sections. (**E**) Liver TAG measurement. (**F**) qPCR analysis of hepatic gene expression. (**G**) Immunoblotting of total liver lysates. (**H**) A schematic model of hepatoprotective effects of THEMIS during MASH. Data in **B**, **C**, **E**, and **F** represent mean ± SEM; 2-tailed unpaired Student’s *t* test. *P < 0.05, **P < 0.01. Scale bars: 100 μm (**D**).

We next examined whether targeted THEMIS overexpression in senescent hepatocytes is sufficient to confer protective effects during diet-indued MASH. We constructed a double-floxed inverse open reading frame (DIO) AAV vector (AAV-TBG-DIO-THEMIS) to conditionally overexpress THEMIS in p21-high senescent hepatocytes using a recently described *p21*-Cre transgenic model (54). We transduced *p21*-Cre–transgenic mice fed MASH diet for 12 weeks with AAV control or AAV-TBG-DIO-THEMIS, followed by tamoxifen administration (Figure 8A). Mice with AAV-mediated THEMIS overexpression in *p21*^hi^ hepatocytes displayed lower liver weight and improved liver injury and hepatic steatosis (Figure 8, B–E), along with reduced hepatic fibrosis, as revealed by Sirus red staining (Figure 8D). Importantly, hepatic SA-β-Gal staining, P21 protein levels, expression of daHep markers (*Nrg1*, *Anxa2*), and ERK phosphorylation were reduced by THEMIS overexpression in *p21*^hi^ hepatocytes (Figure 8, D, F, and G). Taken together, these data illustrate that AAV-mediated overexpression of THEMIS in *p21*^hi^ hepatocytes was sufficient to confer protection against diet-induced MASH in mice by modulating hepatocyte senescence (Figure 8H).

## Discussion

Single-cell transcriptomic analyses of both healthy and diseased hepatic tissues have elucidated previously unrecognized dimensions of cellular heterogeneity and dynamic reprogramming within the liver. A defining aspect of hepatocyte spatial heterogeneity is metabolic zonation along the portal-central axis, which underpins the transcriptional and functional specialization required for the execution of diverse metabolic and secretory processes. However, the extent of hepatocyte heterogeneity beyond classical zonation remains incompletely characterized. In this study, we performed snRNA-seq analysis to interrogate healthy and diet-induced MASH mouse livers. We identified a distinct subpopulation of MASH-associated hepatocytes characterized by elevated expression of marker genes such as *Nrg1*, *Anxa2*, and *Gdf15*. This subpopulation exhibits a transcriptional profile reminiscent of a hepatocyte subtype previously implicated in liver injury and tumorigenesis. Notably, ANXA2^+^ migratory hepatocytes are induced following acetaminophen-mediated hepatic injury, facilitating wound closure and regenerative processes (39). Furthermore, the daHep subpopulation demonstrates an enrichment for copy number variations, implicating it in hepatocarcinogenesis (22, 23). The emergence of daHep in MASH livers strongly suggests that its induction constitutes a shared cellular response to both acute and chronic hepatic injury.

Transcriptomic profiling of the daHep cluster revealed a notable enrichment for senescence-associated gene expression. Nevertheless, hepatocytes within the daHep cluster and those exhibiting high senescence scores are not entirely congruent, indicating partial overlap and suggesting a potential ontological relationship within the context of MASH pathogenesis. Cellular senescence has been increasingly recognized as a pathogenic driver in metabolic disorders, including insulin resistance and MASH (28–30). In this study, we identified THEMIS as a critical hepatoprotective factor that mitigates hepatocyte senescence, daHep induction, and disease progression. While THEMIS expression is abundant in T lymphocytes, both our single-cell RNA-seq and snRNA-seq analysis revealed that THEMIS is markedly upregulated in MASH hepatocytes but not T cells. Importantly, this induction of hepatic THEMIS expression is conserved between murine and human MASH. THEMIS expression is robustly induced by fatty acid exposure, but not by inflammatory cytokines, in cultured hepatocytes, implicating lipid overload as a potential trigger in MASH livers. In support of this, THEMIS expression was most robustly induced in hepatocytes located in zones 2 and 3, which are more prone to developing hepatic steatosis. Both global and hepatocyte-specific ablation of THEMIS exacerbated diet-induced MASH phenotypes in mice, including hepatocellular injury, inflammation, and liver fibrosis. Moreover, AAV-mediated overexpression of THEMIS ameliorated MASH pathology in transduced mice. These findings underscore the hepatoprotective role of THEMIS and establish its essential function in orchestrating adaptive hepatic responses during the pathogenesis of metabolic liver disease.

THEMIS confers hepatoprotective effects via several nonmutually exclusive mechanisms. Gain- and loss-of-functions studies revealed a substantial role of THEMIS in mitigating diet-induced hepatic steatosis. THEMIS inactivation exacerbated fat accumulation in the liver, whereas its overexpression markedly improved diet-induced hepatic steatosis. Supporting these observations, snRNA-seq analyses indicated THEMIS deficiency is associated with upregulation of genes involved in hepatic lipogenesis and downregulation of genes involved in FAO in hepatocytes. A second major function of THEMIS in the context of MASH is the suppression of hepatocyte senescence and daHep induction in the liver. Genetic ablation of THEMIS resulted in a pronounced increase in expression of p21, a canonical marker of cellular senescence, within hepatocytes. Furthermore, analysis using senescence-related gene sets indicates a higher prevalence of hepatocytes with elevated senescence scores in HKO mice. THEMIS also robustly inhibits the induction of hepatic genes characteristic of daHep during diet-induced MASH. Given the established contribution of hepatocyte senescence and daHep induction to liver disease pathogenesis, these findings support the concept that suppression of these processes is protective against disease progression. A recent study demonstrated that daHep exhibit increased genome instability and may contribute to liver tumorigenesis (23). Whether hepatic THEMIS plays a role in MASH-associated hepatocellular carcinoma remains to be fully explored. Beyond cell-autonomous effects, hepatocyte THEMIS signaling exerts diverse non-cell-autonomous influences on the NPC compartment of the MASH liver microenvironment. These include modulation of HSC activation state and alterations in macrophage composition, as revealed by snRNA-seq analysis. The exact mechanisms that link hepatic THEMIS signaling to macrophage recruitment and polarization and HSC activation remain an important unresolved question. Collectively, these results demonstrate that THEMIS mediates hepatoprotection through both hepatocyte-intrinsic and -extrinsic mechanisms.

THEMIS regulates proximal T cell receptor signaling in thymocytes by forming a ternary complex with GRB2 and SHP1/2, thereby modulating downstream ERK activation (47–50). In the context of MASH, we found that THEMIS inactivation resulted in aberrant ERK activation in the liver, whereas AAV-mediated overexpression of THEMIS suppressed this pathway. This inhibitory effect of THEMIS on ERK activation is cell-autonomous within hepatocytes. Mechanistically, THEMIS binds with GRB2 in MASH liver and suppresses RAS activity, thereby reducing ERK activation. ERK signaling plays a complex role in the regulation of senescence; while it can suppress cellular senescence under some conditions, constitutive ERK activation may promote senescence induction (55, 56). In cultured hepatoma cells, ERK was activated during senescence induction in response to Dox, and adenovirus-mediated THEMIS overexpression attenuated both ERK activation and Dox-induced hepatocyte senescence. These findings indicate that THEMIS acts as an upstream regulator of ERK signaling cascade to restrain maladaptive hepatocyte senescence. Although recent studies have implicated BMP4/Gremlin 1–mediated modulation of YAP/TAZ activity in the regulation of hepatocyte senescence (35), the expression of Bmp4 and Grem1 was largely unaltered by THEMIS inactivation, suggesting that these pathways likely operate independently. The robust inhibitory effects of THEMIS on MASH-associated hepatocyte senescence highlight its potential as a therapeutic target for the treatment of metabolic liver disease.

## Methods

### Sex as a biological variable

Male and female liver samples were used for gene expression analysis. We performed in vivo metabolic studies using male mice because female mice are resistant to the development of key features of human MASH following MASH diet feeding. Primary hepatocytes were isolated from both male and female mice in this study.

### Plasmid construction

*Themis* transcript variant 1 (NM_178666.6), subcloned into our lab-modified AAV-TBG vector; pAdTrack-CMV expression vector (Addgene); and AAV-TBG-DIO vector, provided by Liangyou Rui, University of Michigan. Flag and HA double-tagged THEMIS was subcloned into AAV-TBG vector.

### Diet-induced MASH mouse models

To induce MASH, mice were fed a MASH diet containing 40 kcal% fat, 20 kcal % fructose, and 2% cholesterol (D09100310, Research Diets) at the age of 10–12 weeks. Plasma and tissue were harvested after 3 or 5 months of MASH diet feeding for further analysis. For AAV administration studies, mice were fed a MASH diet for 12 weeks and then tail vein injected with AAV8-Themis, AAV8-Cre, or AAV8-Flag-HA-Themis at the dose of 1 × 10^11^ genome copies/mouse. The mice were continuously fed a MASH diet for 8 weeks or 9 days after AAV8-Flag-HA-Themis administration until analysis. *p21*-Cre mice were fed MASH diet for 12 weeks and then tail vein injected with AAV-TBG-DIO-Themis at the dose of 1 × 10^11^ genome copies/mouse, followed by tamoxifen (Sigma, T5648) at the dose of 20 mg/mL (corn oil) intraperitoneal injection for consecutive 4 days. Mice were continued on MASH diet for 4 weeks until analysis.

### In vitro hepatocyte treatment

Primary hepatocytes were isolated from mouse livers using collagenase digestion as previously described (57). Diseased adult human hepatocytes were obtained from the Organ Processing Hub and the Center for Transcriptional Medicine, Department of Pathology, University of Pittsburgh. The Institutional Review Board at the University of Pittsburgh approved our protocol and has given the Not Human Research Determination (IRB# STUDY24020093). Hepatocytes were isolated using a 3-step collagenase digestion technique as previously described (58). Cell viability was assessed after isolation using trypan blue exclusion, and only cell preparations with viability >75% were used for the analysis. Adenoviral infection was performed after 24 hours of isolation, followed by either harvest or treatment with SHP099 (MedChemExpress, HY-100388) for 4 hours. After 2 hours of isolation, mouse primary hepatocytes were treated with mouse EGF recombinant protein (Thermo Fisher, PMG8044) at indicated time points. Hepa1 cells (ATCC, CRL-1830) were transduced by adenovirus and then treated with Dox (Cayman, 15007), MEK inhibitor trametinib (MedChemExpress, HY-10999)], or the combination.

### Bulk RNA-seq and data analysis

Three liver samples from each group (WT and Themis-KO) were used for bulk RNA-seq experiments. Total RNA was extracted from frozen liver tissues with Trizol reagent (TRZ-100, Alkali Scientific) following manufacturer’s protocol. Sequencing libraries for mRNA were generated with NEBNext Poly(A) mRNA Magnetic Isolation Module (NEB, E7490) and NEBNext UltraExpress RNA Library Prep Kit (NEB, E3330) and were paired-end sequenced with 151 bp read length on an Illumina NovaSeq 6000 instrument. The raw sequencing reads were mapped to the reference genome GRCm38 (ENSEMBL) using STAR (v2.7.8a) (59). Gene count estimation for each library was performed using RSEM (v1.3.3) (60). Downstream differential gene expression analysis between genotypes was conducted using DESeq2 (v1.40.0) (61) in the R environment. Cell type deconvolution analysis from the bulk RNA-seq data was conducted with BayesPrism (62) (v2.2.2) using cell type–specific marker genes identified in the snRNA-seq dataset (this study) and a scRNA-seq dataset (GSE119340) as reference.

### Nuclei isolation and snRNA-seq experiment

Liver tissues were snap frozen in liquid nitrogen and kept at –80°C until use for nuclei isolation following a procedure as previously described (21). Briefly, we pooled liver tissue from 4 *Themis* Flox and 4 *Themis* HKO mice, respectively. Approximately 45 mg pooled frozen liver tissue was placed into a 60 mm dish on ice with 1 mL precold salt-Tris (ST) buffer containing 146 mM NaCl, 10 mM Tris-HCl, 1 mM CaCl_2_, 21 mM MgCl_2_, 0.03% Tween-20, 0.01% BSA, and 0.2 U/mL Protector RNase Inhibitor (Sigma, 3335399001) in nuclear-free water. The tissue was finely chopped using Noyes Spring Scissor for 10 minutes on ice, followed by homogenization using glass dounce homogenizer (Kimble, 885300-0001). The homogenate was filtered through a 70 mm cell strainer and transferred to a 50 mL centrifuge tube. The sample volumes were brought up to 5 mL by adding 2 mL wash buffer (ST buffer without Tween-20, BSA and RNase inhibitor). The filtered homogenate was then centrifuged at 500*g* for 5 minutes at 4 in a swinging bucket centrifuge. The pellets were resuspended in precold PBS buffer with 1% fatty acid free BSA and 0.2 U/mL RNase Inhibitor and filtered through a 40 mm cell strainer. The nuclei resuspension was stained by 1 mg/mL propidium iodide (Sigma, P4170) and then underwent fluorescence-activated cell sorting using a BD FACS Discover S8 Cell Sorter (BD Bioscience) with 100 μm nozzle in the Flow Cytometry Core at the University of Michigan. Sorted nuclei were processed for snRNA-seq using the 10X Genomics Chromium Single-Cell 3’ Gene Expression pipeline and subjected to 28 × 151 bp of sequencing on an Illumina NovaSeq X Plus system in the Advanced Genomics Core at the University of Michigan.

### snRNA-seq data analysis

Demultiplex sequencing reads were mapped reference transcriptome mm10-2020-A downloaded from 10x Genomics’ website using Cell Ranger (v8.0.1), excluding intronic reads by setting the include-introns argument to false. The output gene count by cell matrix files were further processed using the Seurat package (v5.1.0) (63) for filtering out cells with fewer than 300 features or genes expressed in fewer than 3 cells and for data integration. Doublets were removed both with DoubletFinder (v2.0.5) (64) and by manual curation. Initial cell clustering was performed using the top 3,000 variable features with principal components 1–15 at a resolution of 0.2, and cell types were manually annotated based on marker genes’ expression. Differential gene expression analyses were performed following the pseudobulking approach using the edgeR package (v4.4.2) (65), and *P* values were adjusted to FDR using Storey’s method (66).

Pathway signature scores were calculated with the scaled normalized gene expression from gene sets representing the pathways using a principal component analysis–based approach. The first principal component was used as the signature score. For senescence signature, we started with the published gene set (40) for cellular senescence with the addition of *Cdkn1a* and *Gdf15*. The gene set was subsequently filtered based on maximum normalized expression level greater than 0.5, exhibiting large variation among hepatocytes and excluding the ones highly expressed in NPC. Gene sets for proliferation, DNL, and FAO were similarly curated. All the resulting genes for each gene set were listed in Supplemental Table 1.

### Immunostaining and tissue histology

Liver tissue was fixed with 4% paraformaldehyde at 4°C with mild shaking overnight, followed by PBS wash twice and then incubated with 30% sucrose in PBS overnight. The liver tissue was embedded in OCT. Frozen sections (10 μm) were permeabilized with 1% Triton X-100 in PBS and then blocked with 5% serum and 2% BSA in PBS, followed by incubation in primary antibody solution overnight at 4°C. The secondary antibody solution was applied at room temperature for 1 hour avoiding light. Sections were mounted in Gold Antifade Mountant (Invitrogen, P36930). Images were captured using Leica SP5 and SP8 Confocal Microscope System.

For histology, liver tissue was fixed with 10% formalin at 4°C with mild shaking overnight, followed by PBS wash and then embedded in paraffin prior to sectioning. H&E staining was performed to assess liver steatosis and immune cell infiltration. Liver fibrosis was assessed by Picrosirius (Sirius) red (Polysciences, 24901) staining of the formalin-fixed, paraffin-embedded liver sections. Results were examined using microscopy (Thermo Fisher, EVOS M5000).

### RNAscope in situ hybridization

RNAscope assay was performed using fresh-frozen liver sections following the user manual of the Advanced Cell Diagnostics RNAscope Multiplex Fluorescent Reagent Kit v2 (UM 323100). Briefly, snap-frozen liver tissue was embedded in cyroembedding medium (OCT). Liver sections were fixed using 4% PFA, and were applied with RNAscope hydrogen peroxide (Advanced Cell Diagnostics, 2018518) and protease IV (Advanced Cell Diagnostics, 2018519). Human *THEMIS* probe (Advanced Cell Diagnostics, 551661), mouse *Nrg1* probe (Advanced Cell Diagnostics, 418181), mouse *Cdkn1a* probe (Advanced Cell Diagnostics, 408551-C2), TSA Vivid fluorophore 570 (Advanced Cell Diagnostics, 323272), and TSA Vivid fluorophore 650 (Advanced Cell Diagnostics, 323273) were used for signal detection. DAPI was then applied to sections before mounting using antifade mounting medium (Invitrogen, P36930). Images were captured on a Leica SP8 confocal microscope.

### Senescence staining

Liver tissue sections were prepared as previously described (67). In brief, fresh liver tissue was immediately embedded in OCT compound and flash-frozen in the clear portion of isopentane (Fisher Scientific, 78-78-4) in a plastic container that sat in the liquid nitrogen. Liver sections were cut at thickness of 12 μm. Cellular senescence in the liver and cell culture were evaluated by SA-β-GAL staining using a commercial kit (Cell Signaling Technology, 9860), following the manufacturer’s instructions. Images were captured using microscopy (Thermo Fisher, EVOS M5000).

### Immunoblotting analysis

Approximately 20 mg liver tissue was homogenized in a lysis buffer containing 50 mM Tris (pH7.5), 130 mM NaCl, 5 mM NaF, 25 mM β-glycerophosphate, 1 mM Na_3_VO_4_, 10% glycerol, 1% Triton X-100, 1 mM dithiothreitol (DTT), and freshly added protease inhibitor cocktail (Roche, 1187358001). Cells were harvested and homogenized with the lysis buffer. The protein concentration was determined using a Bio-Rad kit (5000006). Three-fold SDS loading buffer (6% SDS, 0.03% bromophenol blue, 25% glycerol, 0.25 M Tris-HCl, pH 6.8, and 10 mM DTT) was added to the protein lysates, after which the buffer was mixed and boiled for 5 minutes. Protein lysates were separated using SDS-PAGE and then transferred to the PVDF membrane. The BrightStar Duration HRP Chemiluminescent 2-Component Substrate ECL Kit (Alkali Scientific Inc, XR92) or Clarity Western ECL Substrate (Bio-Rad, 1705060S) was used for signal detection.

### Antibodies

Antibodies used for this study included anti-Themis (Abcam, ab126771), anti-p21(Cell Signaling Technology, 2947), anti-NRG1 (Boster Bio, PA1969), anti-ANXA2 (Cell Signaling Technology, 8235), Hsp90 (Santa Cruz, Sc-13119), anti-phospho-Erk (T202/Y204) (Cell Signaling Technology, 4370), anti-Erk (Cell Signaling Technology, 4695), anti-phospho-p38 (T180/Y182) (Cell Signaling Technology, 9215), anti-phospho-JNK (T183/Y185) (Cell Signaling Technology, 4668), anti-phospho-NF-κB (S536) (Cell Signaling Technology, 3033), anti-phospho-H2A.X contained in the Senescence Marker Antibody Sampler Kit (Cell Signaling Technology, 56062), anti-GRB2 (ABclonal, A19059), anti-Flag (Sigma, F1804), anti-DESMIN (Thermo Fisher, RB-9014-P1), anti-F4/80 (Bio-Rad, MCA497G), anti-CK8 (Developmental Studies Hybridoma Bank, clone ID: TROMA-I).

### Immunoprecipitation

Mouse livers were snap-frozen in liquid nitrogen and homogenized in lysis buffer with protease inhibitor cocktail (Roche, 1187358001) as previously described (68). The supernatants were collected and incubated with anti-Flag M2 beads (Sigma, A2220) overnight at 4°C with gentle rocking. On the following day, Flag beads bound with proteins were washed by centrifugation and Flag peptide was added to beads mixture at 0.2 mg/mL with gentle rocking at room temperature for 30 minutes. After centrifugation, the elutes were incubated with anti-HA agarose beads (Thermo Fisher, 26181) for 4 hours at 4°C with gentle rocking. HA beads bound with proteins were washed by centrifugation, and HA peptide was added to beads mixture at 0.2 mg/mL. The mixtures were then incubated at room temperature for 30 minutes with gentle rocking. The HA elutes were collected and added with 3-fold SDS buffer, after which the elutes were mixed and boiled for 5 minutes for SDS-PAGE and immunoblotting.

### RAS activity assay

The RAS Pull-down Activation Assay Biochem kit (Cytoskeleton, BK008) was used to measure in vivo RAS activity following the manufacturer’s instructions. Briefly, cells were washed once in cold 1X PBS, and then precold lysis buffer was added to resuspend the cells. Lysates were centrifugated at 10,000*g* for 1 minute at 4°C. Supernatants were collected and analyzed for protein concentration using a Bio-Rad kit (5000006). A total of 1 mg protein lysates was incubated with Raf-RBD beads (Cytoskeleton, BK008) for affinity purification of active RAS at 4°C for 1 hour with gentle rocking. After washing and centrifugation, beads were boiled with 3-fold SDS for 2 minutes, followed by immunoblotting using anti-pan RAS antibody included in the kit.

### RNA extraction and gene expression analysis

Total liver or cellular RNA was extracted using the Trizol (Alkali Scientific, TRZ-100) method. RNA samples were reverse transcribed to cDNA using the MMLV-RT method (69). qPCR was conducted for gene expression analysis using the SYBR green (Fisher Scientific, 44-729-20) method.

### Plasma assay

Plasma AST and ALT levels were measured using commercial kits from Teco Diagnostics (A524-150) and Wako (995-34791), respectively. Measurement followed the manufacturer’s instructions.

### Flow cytometry

Cell isolation was conducted as previously described (21). Briefly, ~1.2 g liver tissue was ground and passed through a 100 μm cell strainer in FACS buffer (1X PBS containing 1% FBS and 1 mM EDTA). After centrifugation at 50*g* for 5 minutes to remove hepatocytes pellet, the cell suspension was centrifugated at 500*g* to obtain NPC pellets. Mixed solution of FACS buffer and 50% Optiprep solution (Serumwerk, 1893) at the ratio 1:1 was used to resuspend the NPC pellets, followed by centrifugation at 1,500*g* for 20 minutes with no break. The middle layer of floating cells was collected, washed, and counted prior to antibody staining. Approximately 1 × 10^6^ liver cells were stained with Pacific Blue anti-mouse CD45 (BioLegend, 103126), PE/Cyanine7 anti-mouse F4/80 (BioLegend, 123114), and PerCP/Cyanine5.5 anti-mouse CD11b (BioLegend, 101227). Cell samples were analyzed using an Attune NXT4 Flow Cytometer at the MCDB research core facility at the University of Michigan. Data were analyzed using Flowjo (https://www.flowjo.com/).

### Statistics

Data in bar plots represent as mean ± SEM. All data were assessed for homogeneity of variance by Levene’s test. The normality of data was assessed by Shapiro-Wilk test in R environment. For normally distributed data with equal variance, the differences between groups were determined by 1-way ANOVA followed by post hoc Tukey’s test or 2-tailed unpaired Student’s *t* test. All statistical tests were performed using R.

### Study approval

#### Human liver biopsies.

Human liver samples were obtained from the liver Tissue Cell Distribution System at the University of Minnesota, as previously described (70). Both male and female individuals were included, with the average age of 55.8 and 52.5 years old for patients without MASH and patients with MASH (cohort 1) and 56.1 and 40 years old for patients without MASH and patients with MASH (cohort 2). The Institutional Review Board at the University of Pittsburgh approved our protocol and has given the Not Human Research Determination (IRB# STUDY24020093).

#### Animal studies.

All animal studies described in this paper were conducted following the approval of protocols by the Institutional Animal Care and Use Committee at the University of Michigan. Mice were housed in specific pathogen–free facilities under 12-hour light/dark cycles with free access to food and water. WT C57BL/6J mice were purchased from The Jackson Laboratory (JAX 000664). *Themis*-KO (43) and *Themis* Flox mice were provided by Paul E. Love (NIH). *Themis* HKO mice were generated by crossing *Alb-*Cre mice with the *Themis* Flox mice. *p21*-Cre mice were provided by Dr. Ming Xu (University of Minnesota). Samples sizes were determined based on contemporary work in the literature (14, 46, 70). Mice with the same genotype were randomly assigned to different treatments. Mice with wounds due to fighting or growth retardation due to malocclusion were excluded from experiments.

### Data availability

The raw high-throughput sequencing data were deposited into the NCBI Gene Expression Omnibus (GEO) database under the identifiers GSE300143, GSE300162, and GSE300209. Data points can be accessed from the Supporting Data Values file.

## Author contributions

JDL and XQ conceived the project and designed research. XQ, Y Lu, YT, LZ, Y Lee, ZM, ZC, FP, and SL performed the experiments and analyzed the data. XQ, YL, and JDL performed sequencing data analysis. LAPF, ZH, and ASG provided human primary hepatocytes for treatment studies. JDL, XQ, and YL wrote the manuscript.

## Conflict of interest

The authors have declared that no conflict of interest exists.

## Funding support

This work is the result of NIH funding, in whole or in part, and is subject to the NIH Public Access Policy. Through acceptance of this federal funding, the NIH has been given a right to make the work publicly available in PubMed Central.

- NIH (DK102456, DK136177 to JDL).
- American Heart Association Career Development Award to LZ.
- University of Michigan Rackham Predoctoral Fellowship to ZM.
- NIH P30DK020572 to the University of Michigan Diabetes Research Center.
- NIH Cancer Center Support grant P30CA046592 to the University of Michigan Comprehensive Cancer Center.

## Supplementary Material

Supplemental data

Unedited blot and gel images

Supporting data values

## Version 1 05/01/2026

Electronic publication
