# Supplementary material for: THEMIS attenuates MASH by suppressing disease-associated hepatocyte induction and hepatocyte senescence in mice
Source: J Clin Invest. 2026 May 1;136(9):e199303. doi: 10.1172/JCI199303 (PMC13132366; doi:10.1172/JCI199303)
Supplement: Supplemental data [file jci-136-199303-s135.pdf]

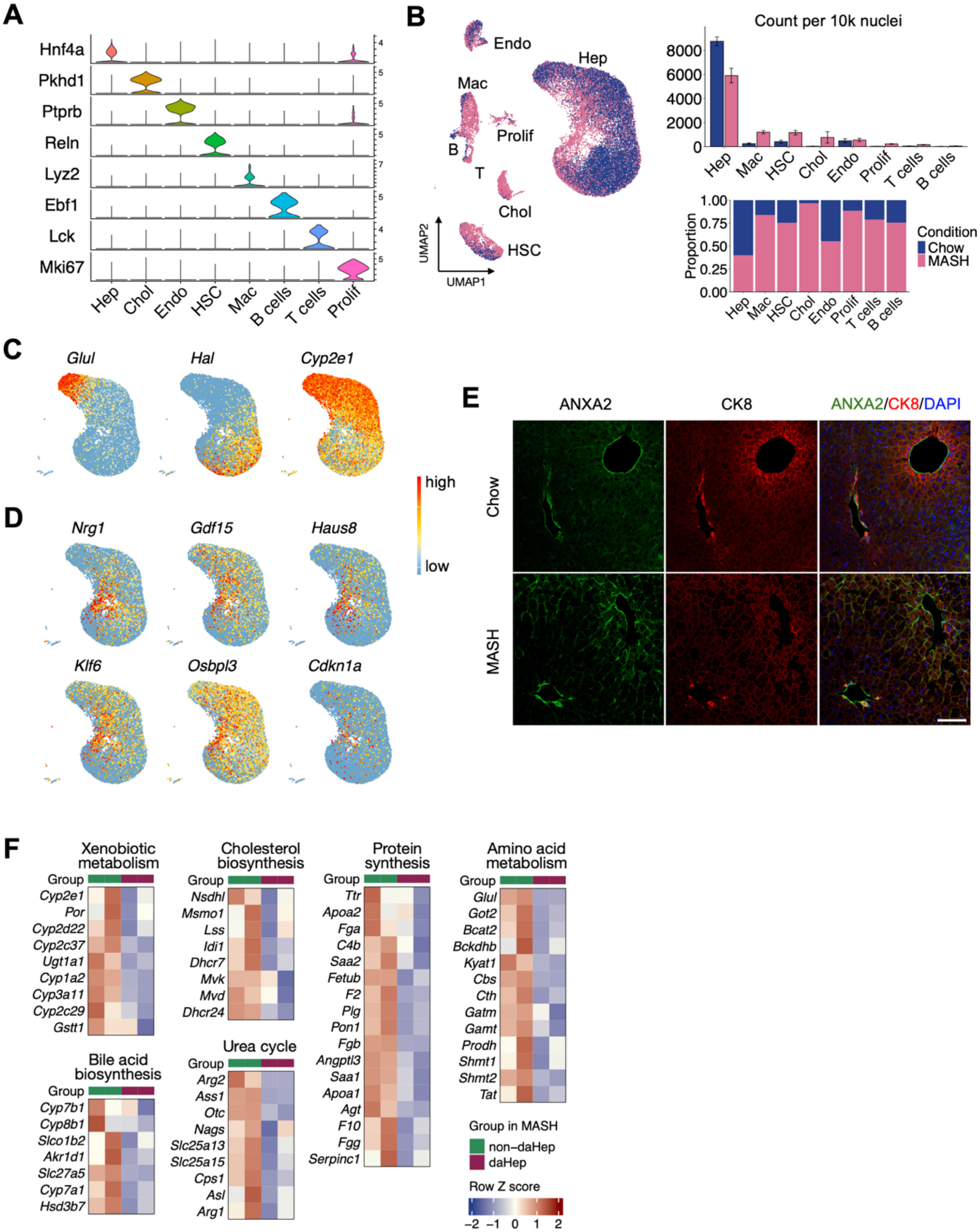

A
Hnf4a
Pkhd1
Ptprb
Reln
Lyz2
Ebf1
Lck
Mki67
Hep
Chol
Endo
HSC
Mac
B cells
T cells
Prolif
B
Endo
Hep
Mac
Prolif
B
T
Chol
HSC
UMAP2
UMAP1
Count per 10k nuclei
8000
6000
4000
2000
0
Proportion
1.00
0.75
0.50
0.25
0.00
Condition
Chow
MASH
C
Glul
Hal
Cyp2e1
high
low
D
Nrg1
Gdf15
Haus8
Klf6
Osbpl3
Cdkn1a
E
ANXA2
CK8
ANXA2/CK8/DAPI
Chow
MASH
F
Xenobiotic metabolism
Group
Cyp2e1
Por
Cyp2d22
Cyp2c37
Ugt1a1
Cyp1a2
Cyp3a11
Cyp2c29
Gstt1
Bile acid biosynthesis
Group
Cyp7b1
Cyp8b1
Slco1b2
Akr1d1
Slc27a5
Cyp7a1
Hsd3b7
Cholesterol biosynthesis
Group
Nsdhl
Msmo1
Lss
Idi1
Dhcr7
Mvk
Mvd
Dhcr24
Urea cycle
Group
Arg2
Ass1
Otc
Nags
Slc25a13
Slc25a15
Cps1
Asl
Arg1
Protein synthesis
Group
Ttr
Apoa2
Fga
C4b
Saa2
Fetub
F2
Plg
Pon1
Fgb
Angptl3
Saa1
Apoa1
Agt
F10
Fgg
Serpinc1
Amino acid metabolism
Group
Glul
Got2
Bcat2
Bckdhb
Kyat1
Cbs
Cth
Gatm
Gamt
Prodh
Shmt1
Shmt2
Tat
Group in MASH
non−daHep
daHep
Row Z score
−2 −1 0 1 2

Supplementary Figure 1. Healthy and diet-induced MASH livers. (A) Stacked violin plot showing normalized expression of cell type-specific marker gene for each cluster. (B) UMAP and bar plots visualizing the proportion of nuclei from Chow and MASH samples. (C-D) Feature plots showing gene expression patterns in all hepatocytes. (E) Immunofluorescence staining for ANXA2 and CK8 of liver sections. (F) Heatmaps of genes associated with essential hepatic pathways grouped by non-daHep and daHep in MASH livers. Scale bars in (E) represent 100 μm.

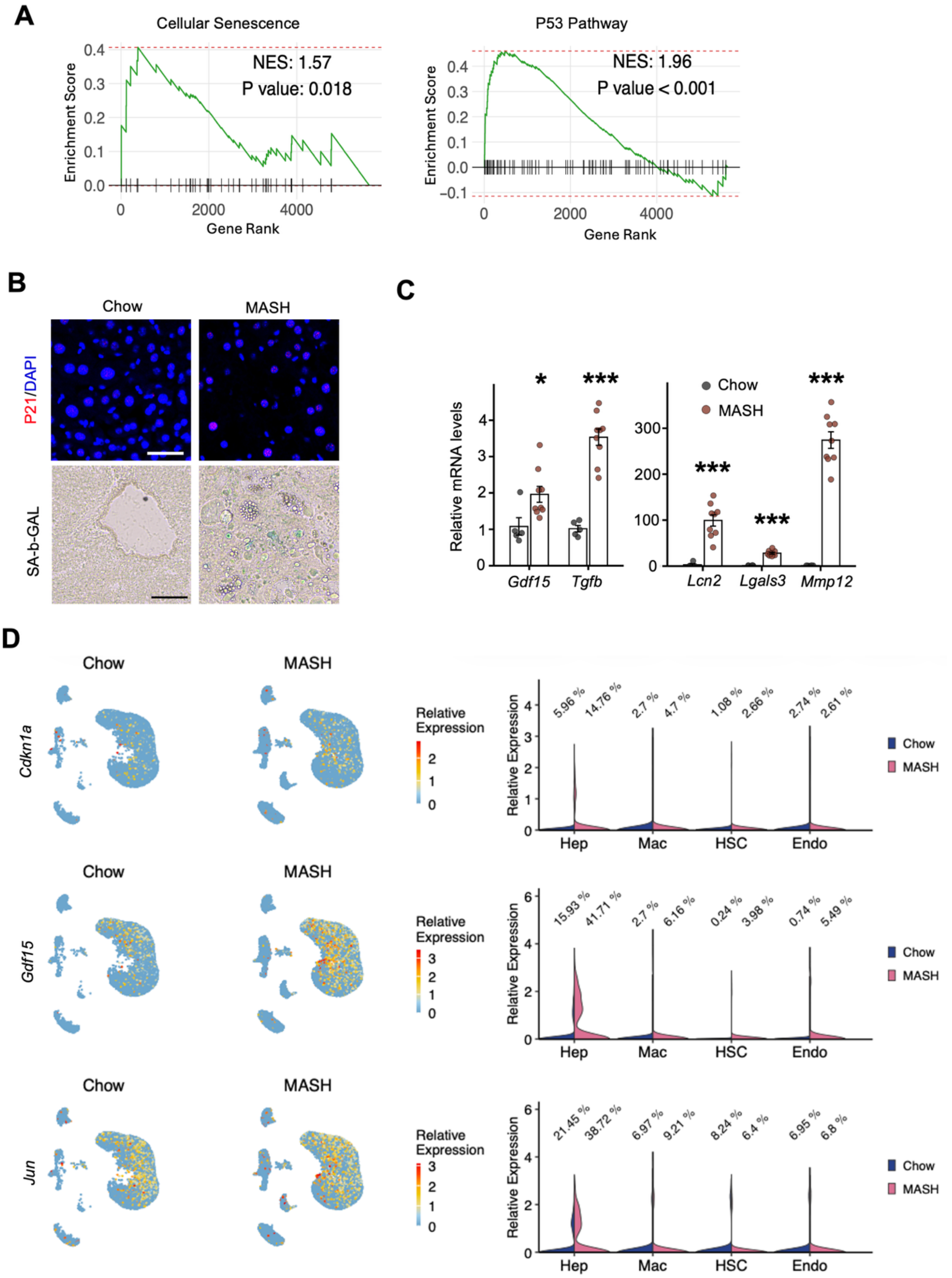
A
Cellular Senescence
NES: 1.57
P value: 0.018
Enrichment Score
Gene Rank
P53 Pathway
NES: 1.96
P value < 0.001
Enrichment Score
Gene Rank
B
Chow
MASH
P21/DAPI
SA-b-GAL
C
Relative mRNA levels
Gdf15
Tgfb
Lcn2
Lgals3
Mmp12
Chow
MASH
D
Chow
MASH
Cdkn1a
Gdf15
Jun
Relative Expression
5.96 %
14.76 %
2.7 %
4.7 %
1.08 %
2.66 %
2.74 %
2.61 %
15.93 %
41.71 %
2.7 %
6.16 %
0.24 %
3.98 %
0.74 %
5.49 %
21.45 %
38.72 %
6.97 %
9.21 %
8.24 %
6.4 %
6.95 %
6.8 %
Hep
Mac
HSC
Endo

Supplementary Figure 2. Cellular senescence is induced during MASH progression. (A) Gene sets enrichment analysis (GSEA) between daHep versus non-daHep cells in MASH highlighting Cellular Senescence pathway and P53 pathway are among the top up-regulated pathways. (B) Immunofluorescence staining for P21 and SA-β-GAL staining of Chow and MASH liver sections. (C) qPCR analysis of senescence-associated gene expression. (D) Feature plots and violin plots showing senescence-associated gene expression in hepatocytes and non-parenchymal cells.

Data in (C) represent mean ± SEM; two-tailed unpaired Student's *t* test. $*P < 0.05$ and $***P < 0.001$. Scale bars in (B) represent 50 μm.

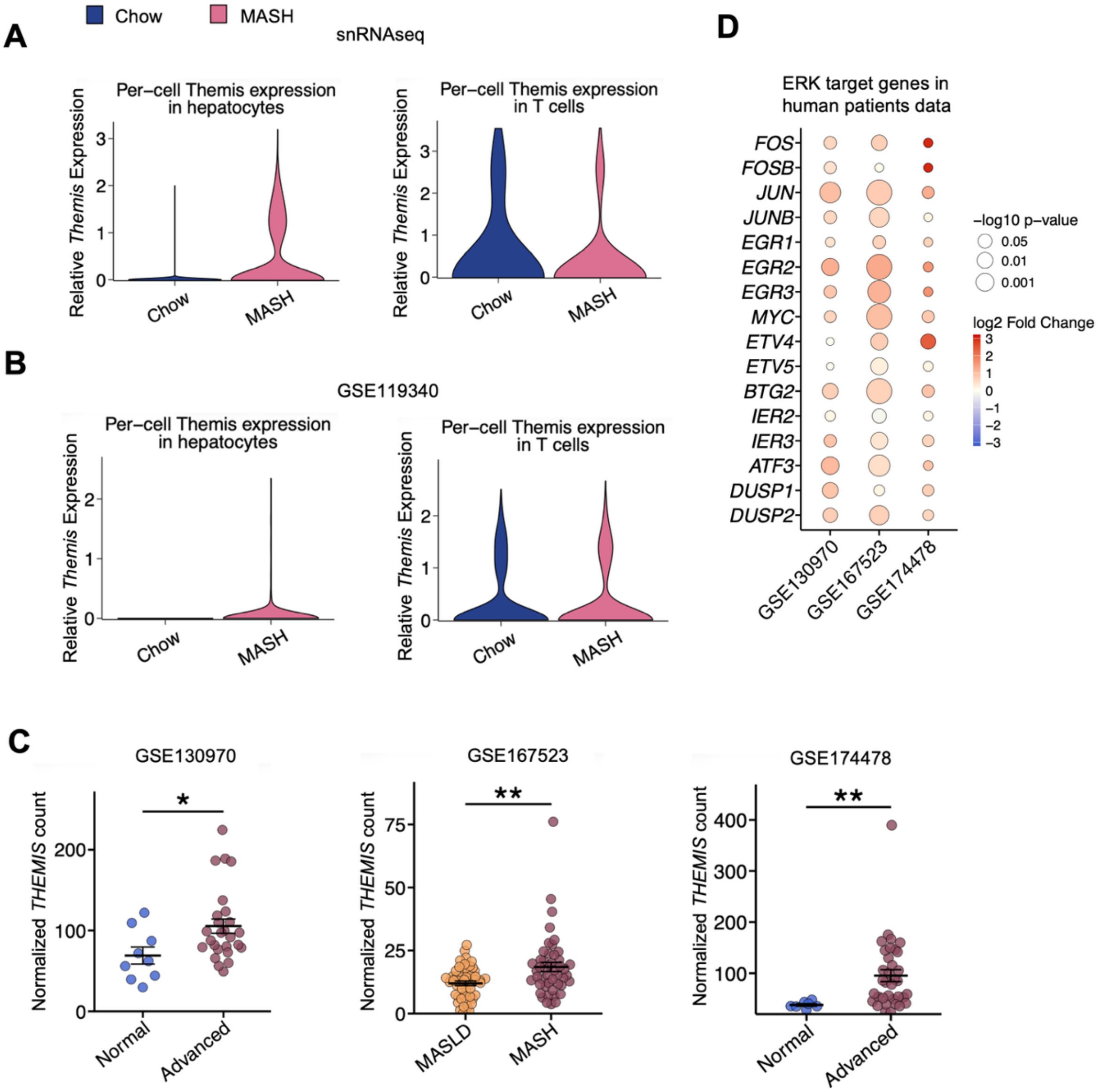

A
Chow
MASH
snRNAseq
Per−cell Themis expression in hepatocytes
Per−cell Themis expression in T cells
Relative Themis Expression
B
GSE119340
C
GSE130970
GSE167523
GSE174478
Normalized THEMIS count
Normal
Advanced
MASLD
D
ERK target genes in human patients data
FOS
FOSB
JUN
JUNB
EGR1
EGR2
EGR3
MYC
ETV4
ETV5
BTG2
IER2
IER3
ATF3
DUSP1
DUSP2
−log10 p−value
log2 Fold Change

Supplementary Figure 3. Induction of Themis expression and ERK activation in MASH livers. (A-B) Violin plots showing *Themis* expression in hepatocytes and T cells using chow and MASH single-nucleus RNAseq dataset (A) and published single-cell RNAseq dataset (B). (C-D) Analysis of published bulk RNAseq datasets showing *Themis* expression (C) and ERK target gene expression (D) in human non-MASH, MASLD and MASH patients' livers. Data represent mean ± SEM; two-tailed unpaired Student's *t* test. $*P < 0.05$ and $**P < 0.01$.

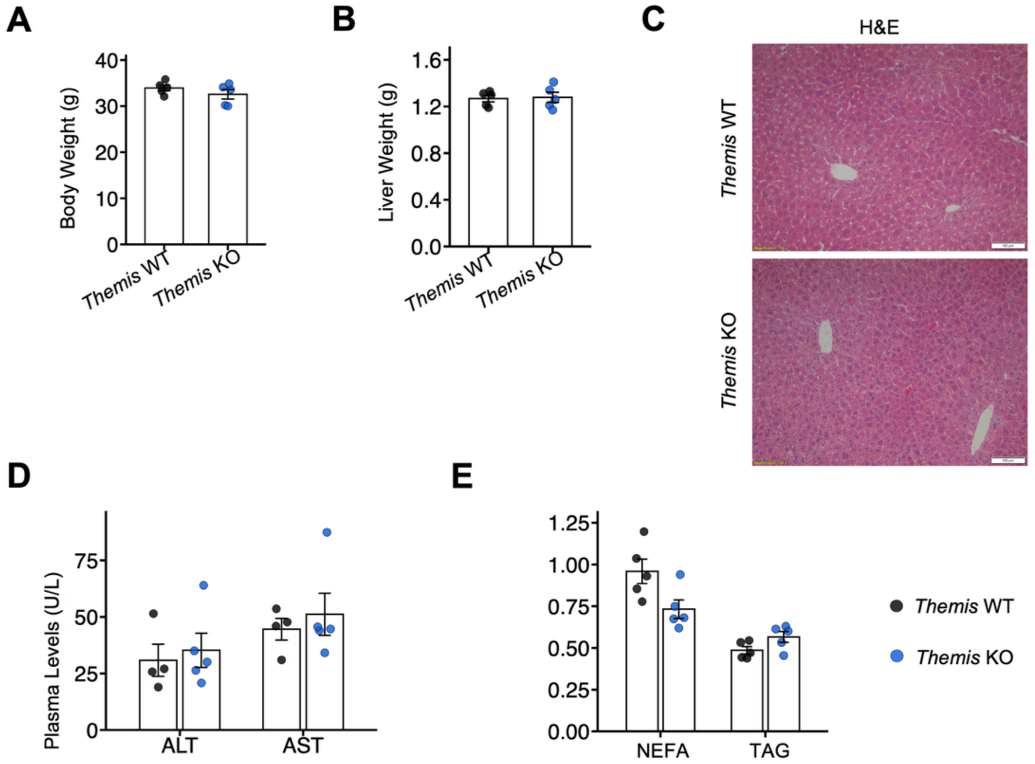

Supplementary Figure 4. Physiological characterization of 9-month-old *Themis* WT and *Themis* KO mice fed on Chow diet. (A) Bar plot of body weight. (B) Bar plot of liver weight. (C) Histology of liver by H&E staining on liver sections. (D) Bar plot of plasma ALT and AST measurement. (E) Bar plot of plasma non-esterified fatty acids (NEFA; mmol/L) and plasma triacylglycerols (TAG; mg/mL) measurements. Values represent mean ± SEM; two-tailed unpaired Student's *t* test.

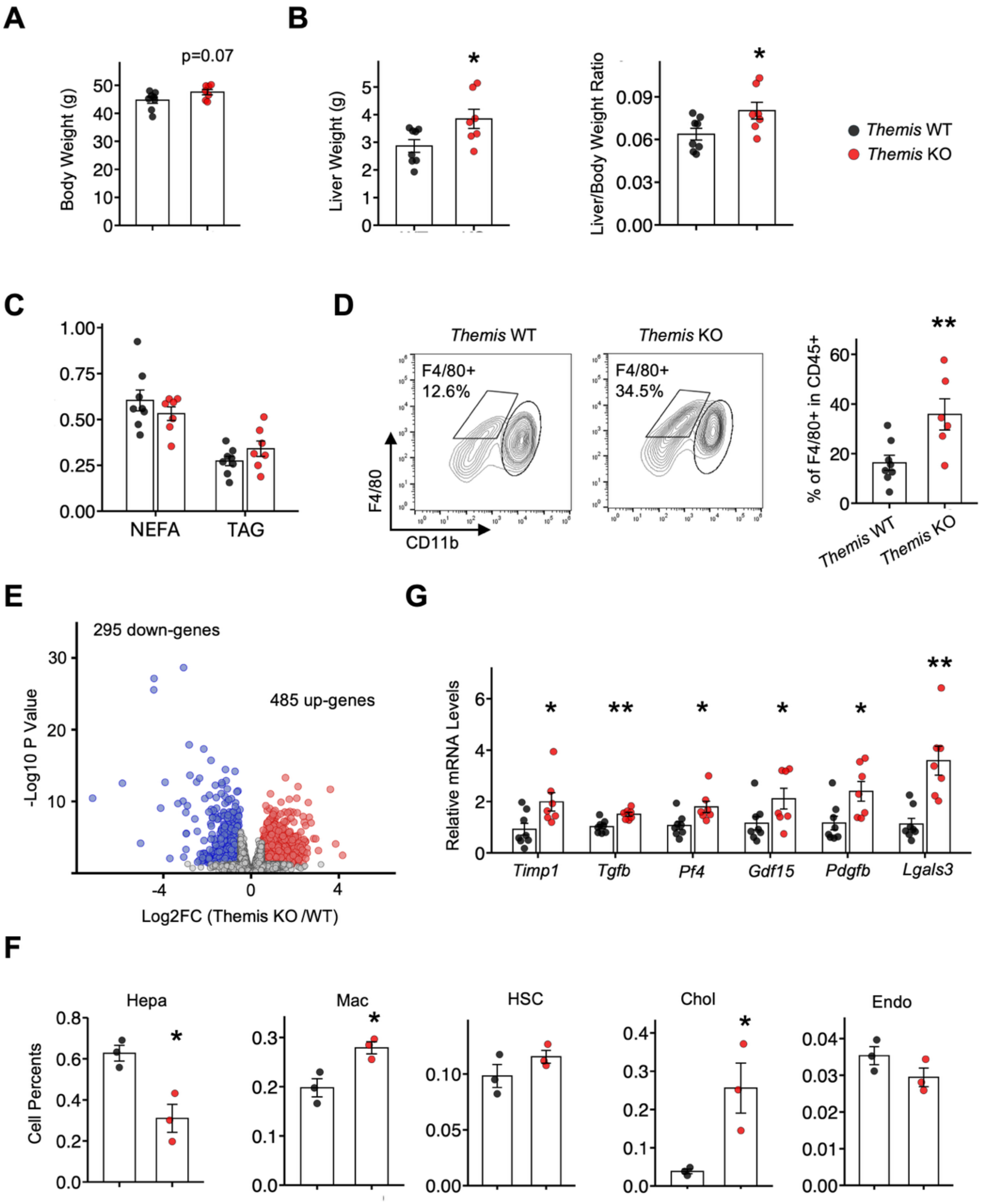
A
B
C
D
E
F
G
p=0.07
Body Weight (g)
Liver Weight (g)
Liver/Body Weight Ratio
Themis WT
Themis KO
NEFA
TAG
F4/80+
12.6%
34.5%
F4/80
CD11b
% of F4/80+ in CD45+
295 down-genes
485 up-genes
-Log10 P Value
Log2FC (Themis KO /WT)
Relative mRNA Levels
Timp1
Tgfb
Pf4
Gdf15
Pdgfb
Lgals3
Cell Percents
Hepa
Mac
HSC
Chol
Endo

Supplementary Figure 5. Whole-body Themis ablation. (A) Body weight of *Themis* WT and *Themis* KO mice fed on MASH diet for 3 months. (B) Liver weight and liver/body weight ratio. (C) Bar plot of plasma NEFA (mmol/L) and plasma TAG (mg/mL) measurements. (D) Flow cytometry analysis of liver macrophages in *Themis* WT and *Themis* KO mice fed on MASH diet, and quantitation of percentage of $F4/80^+$ macrophage among all $CD45^+$ cells. (E) Volcano plot of bulk RNA-seq on liver samples highlighting differentially expressed genes between *Themis* WT and *Themis* KO. (F) Estimated proportion for major liver cell types using deconvoluted bulk RNA-seq data. (G) Gene expression levels in liver measured by qPCR. Values represent mean ± SEM; two-tailed unpaired Student's *t* test. $*P < 0.05$ and $**P < 0.01$.

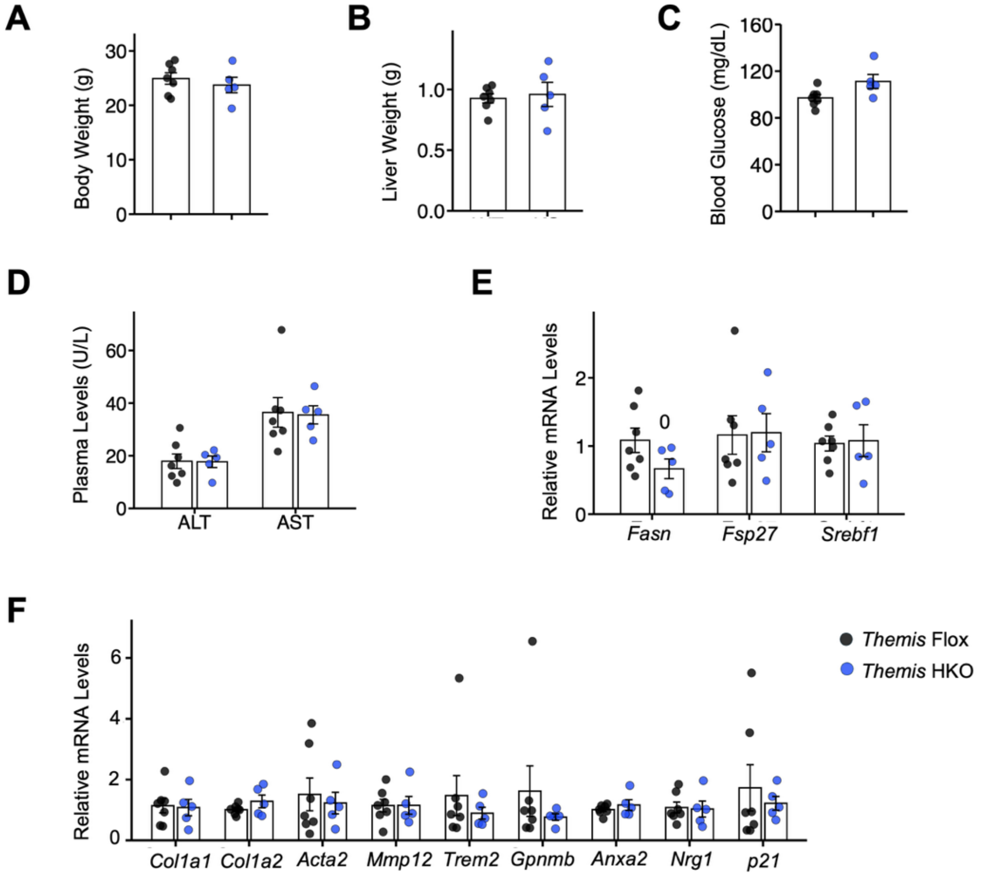

Supplementary Figure 6. Physiological characterization of 3-month-old *Themis* Flox and *Themis* HKO mice fed on Chow diet. (A) Bar plot of body weight. (B) Bar plots of liver weight. (C) Blood glucose after 24-hours fasting. (D) Bar plot of plasma ALT and AST measurement. (E-F) Gene expression levels measured by qPCR. Values represent mean ± SEM; comparisons between genotypes were conducted with two-tailed unpaired Student's *t* test. $* P < 0.05$.

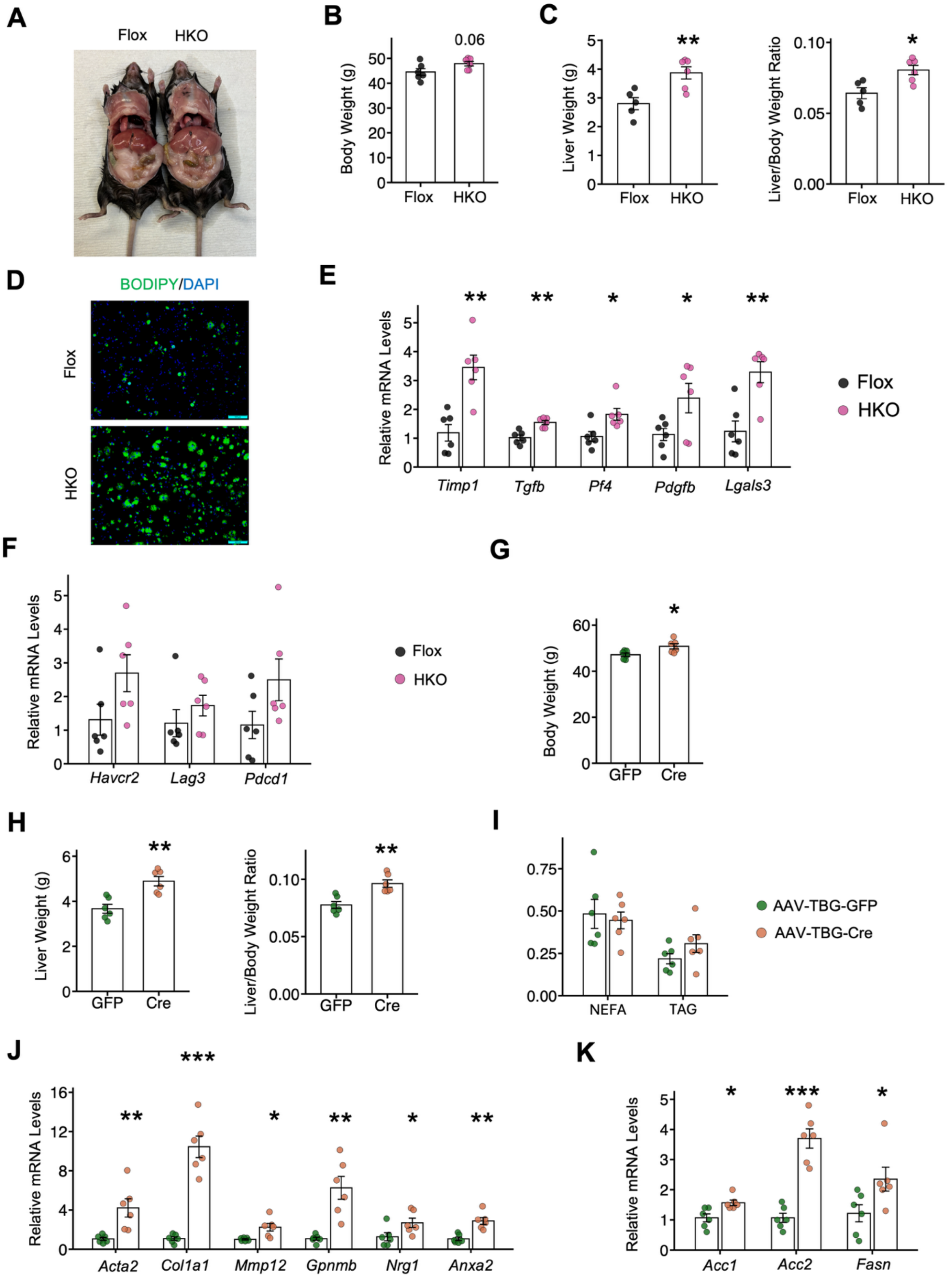
A
Flox
HKO
B
0.06
Body Weight (g)
Flox
HKO
C
**
Liver Weight (g)
*
Liver/Body Weight Ratio
D
BODIPY/DAPI
Flox
HKO
E
** ** * * **
Relative mRNA Levels
Timp1
Tgfb
Pf4
Pdgfb
Lgals3
Flox
HKO
F
Relative mRNA Levels
Havcr2
Lag3
Pdcd1
Flox
HKO
G
*
Body Weight (g)
GFP
Cre
H
**
Liver Weight (g)
**
Liver/Body Weight Ratio
GFP
Cre
I
NEFA
TAG
AAV-TBG-GFP
AAV-TBG-Cre
J
** *** * ** * **
Relative mRNA Levels
Acta2
Col1a1
Mmp12
Gpnmb
Nrg1
Anxa2
K
* *** *
Relative mRNA Levels
Acc1
Acc2
Fasn

Supplementary Figure 7. Hepatocyte-specific THEMIS inactivation. (A-D) *Themis*$^{flox/flox}$ (Flox) (n = 6) and *Themis*$^{Alb\text{-}Cre}$ (HKO) (n = 6) male mice were fed on a MASH diet for 20 weeks. (A) Images of representative *Themis* Flox and HKO mice immediately after dissection. (B) Body weight. (C) Liver weight and liver/body weight ratio. (D) BODIPY staining of primary hepatocytes isolated from *Themis* Flox and HKO mice fed on a MASH diet. (E-F) qPCR analysis of gene expression in *Themis* Flox and Themis HKO livers. (G-K) *Themis* Flox mice were fed on a MASH diet for 12 weeks when those mice were tail-vein injected with either AAV-TBG-GFP (n = 6) or AAV-TBG-Cre (n = 6) and continued the MASH diet for another 8 weeks. (G) Body weight. (H) Liver weight and liver/body weight ratio. (I) Plasma NEFA (mmol/L) and plasma TAG (mg/mL) measurements (J-K) qPCR analysis of gene expression of mice injected with either AAV-TBG-GFP or AAV-TBG-Cre. Values represent mean ± SEM; comparisons between genotypes were conducted with two-tailed unpaired Student's *t* test. $* P < 0.05$, $** P < 0.01$ and $*** P < 0.001$. Scale bars in (D) represent 100 µm.

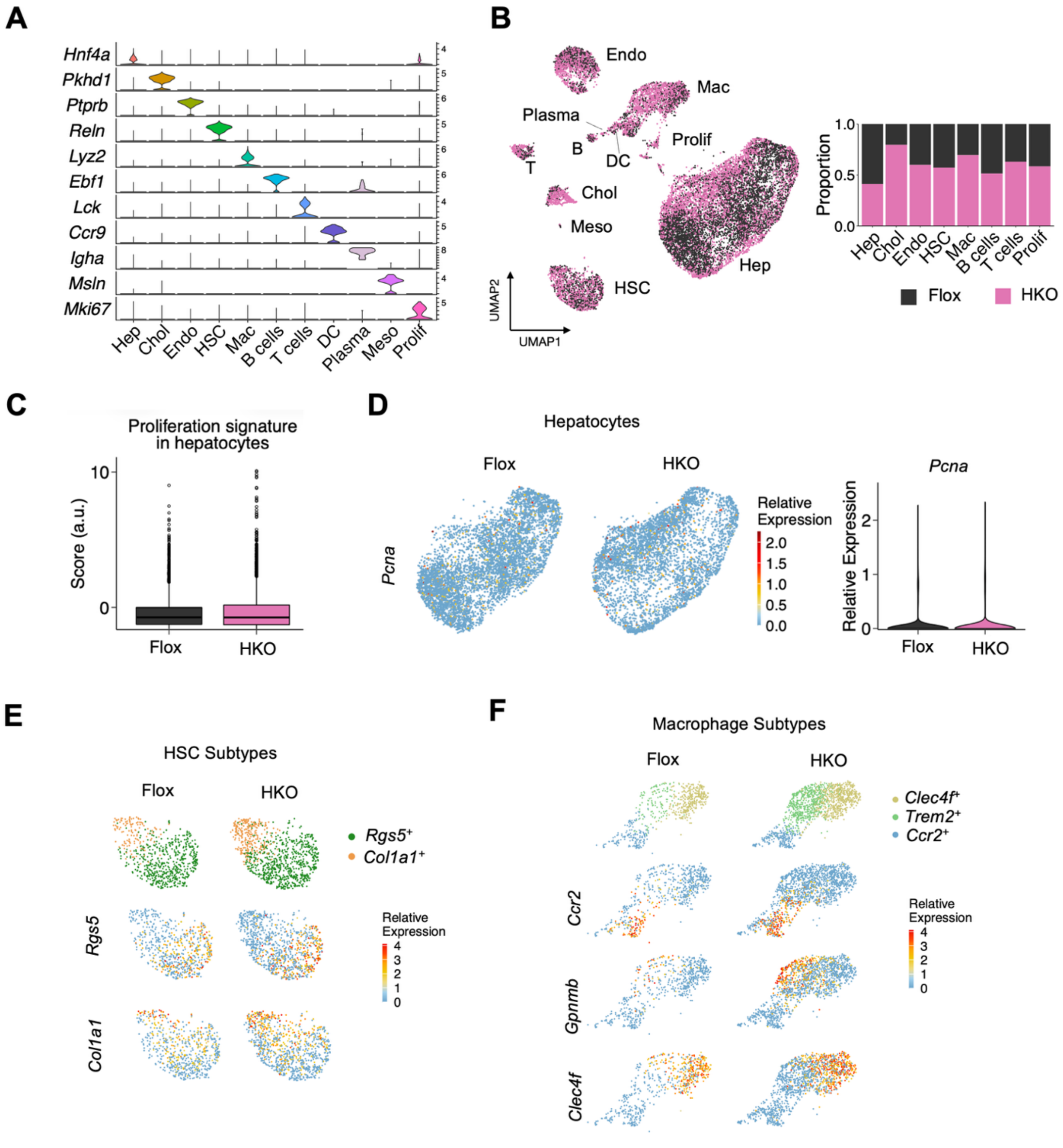

A
Hnf4a
Pkhd1
Ptprb
Reln
Lyz2
Ebf1
Lck
Ccr9
Igha
Msln
Mki67
Hep
Chol
Endo
HSC
Mac
B cells
T cells
DC
Plasma
Meso
Prolif
B
Endo
Mac
Plasma
B
DC
Prolif
T
Chol
Meso
Hep
HSC
UMAP2
UMAP1
Proportion
1.0
0.5
0.0
Flox
HKO
C
Proliferation signature in hepatocytes
Score (a.u.)
10
0
Flox
HKO
D
Hepatocytes
Flox
HKO
Pcna
Relative Expression
2.0
1.5
1.0
0.5
0.0
Pcna
Relative Expression
E
HSC Subtypes
Flox
HKO
Rgs5+
Col1a1+
Rgs5
Col1a1
Relative Expression
F
Macrophage Subtypes
Clec4f+
Trem2+
Ccr2+
Ccr2
Gpnmb
Clec4f
Relative Expression

Supplementary Figure 8. snRNA-seq analysis of *Themis* Flox and HKO livers. (A) Stacked violin plot showing normalized expression of cell type-specific marker gene for each cluster. (B) Visualization of cell clusters grouped by genotype in UMAP coordinates, and a bar plot showing the proportion of nuclei from *Themis* Flox and HKO MASH livers grouped by cell type excluding rare cell types (< 0.5% total nuclei). (C) Analysis of proliferative gene signature scores of *Themis* Flox and HKO hepatocytes. (D) Feature plots and violin plots showing *Pcna* expression in *Themis* Flox and HKO hepatocytes. (E) Subclusters of HSC with marker genes. (F) Subclusters of macrophages with marker genes.

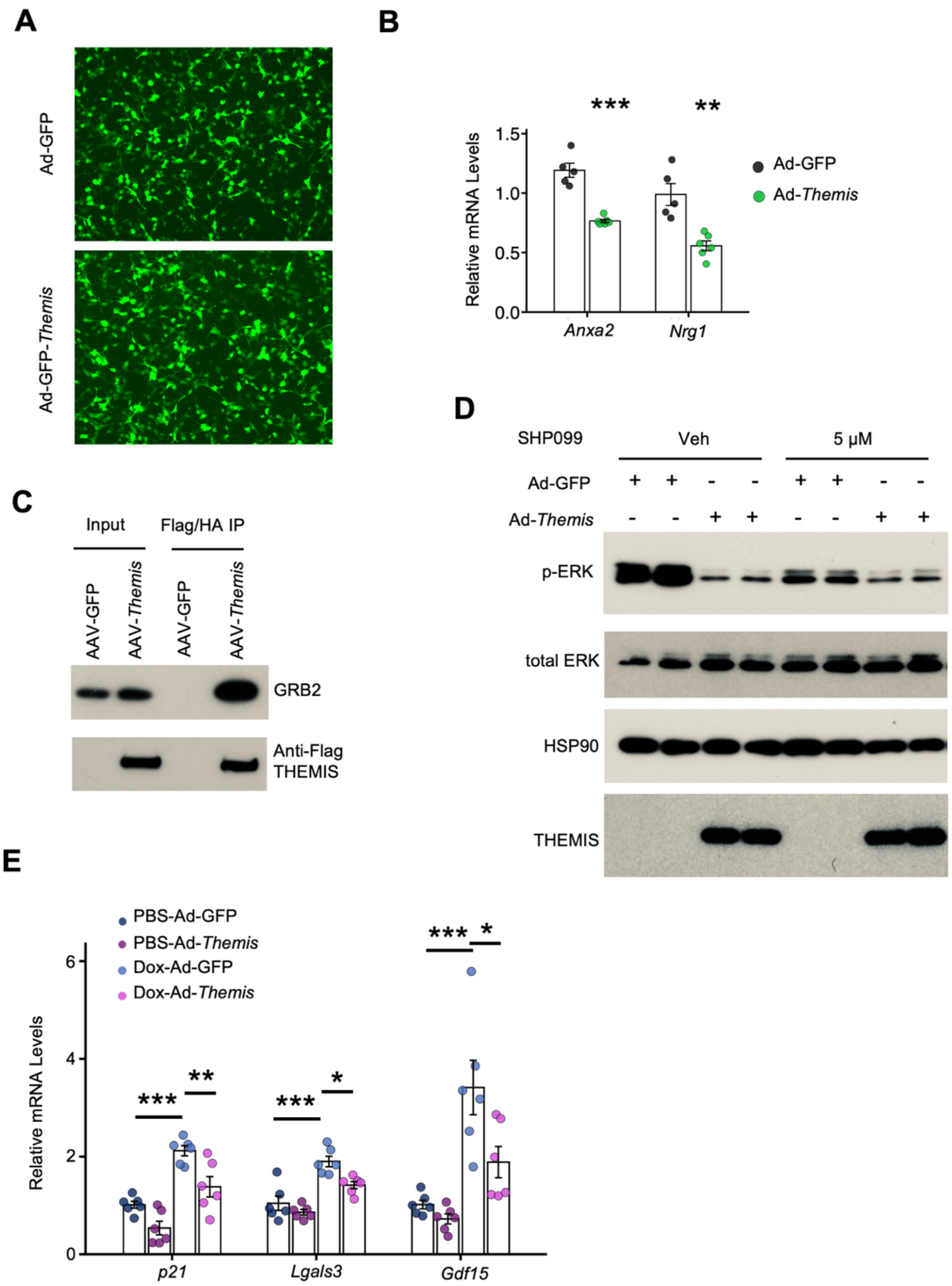
A
Ad-GFP
Ad-GFP-Themis
B
*** **
Relative mRNA Levels
1.5
1.0
0.5
0.0
Anxa2
Nrg1
Ad-GFP
Ad-Themis
C
Input
Flag/HA IP
AAV-GFP
AAV-Themis
AAV-GFP
AAV-Themis
GRB2
Anti-Flag THEMIS
D
SHP099
Veh
5 μM
Ad-GFP + + - - + + - -
Ad-Themis - - + + - - + +
p-ERK
total ERK
HSP90
THEMIS
E
PBS-Ad-GFP
PBS-Ad-Themis
Dox-Ad-GFP
Dox-Ad-Themis
Relative mRNA Levels
6
4
2
0
*** **
*** *
*** *
p21
Lgals3
Gdf15

Supplementary Figure 9. Adenoviral transduction in cultured hepatocytes. (A) Images of Hepa1 cells infected with either Ad-GFP or Ad-GFP-Themis under the GFP channel of a microscope. (B) qPCR analysis of gene expression from primary hepatocytes infected with either Ad-GFP or Ad-Themis. (C) Immunoprecipitation of Flag and HA double tagged THEMIS. (D) Immunoblotting of either Ad-GFP or Ad-GFP-Themis transduced human primary hepatocytes stimulated by SHP099 for 4 hours. (E) Hepa1 cells overexpressing either GFP or Themis were treated with 50nM doxorubicin (Dox) for 3 days. qPCR analysis of gene expression. Data in (B) represent mean ± SEM; two-tailed unpaired Student's *t* test. $**p < 0.01$ and $***p < 0.001$. Data in (E) represent mean ± SEM; One-way ANOVA with post-hoc Tukey's HSD test. $* P < 0.05$, $** P < 0.01$ and $*** P < 0.001$.

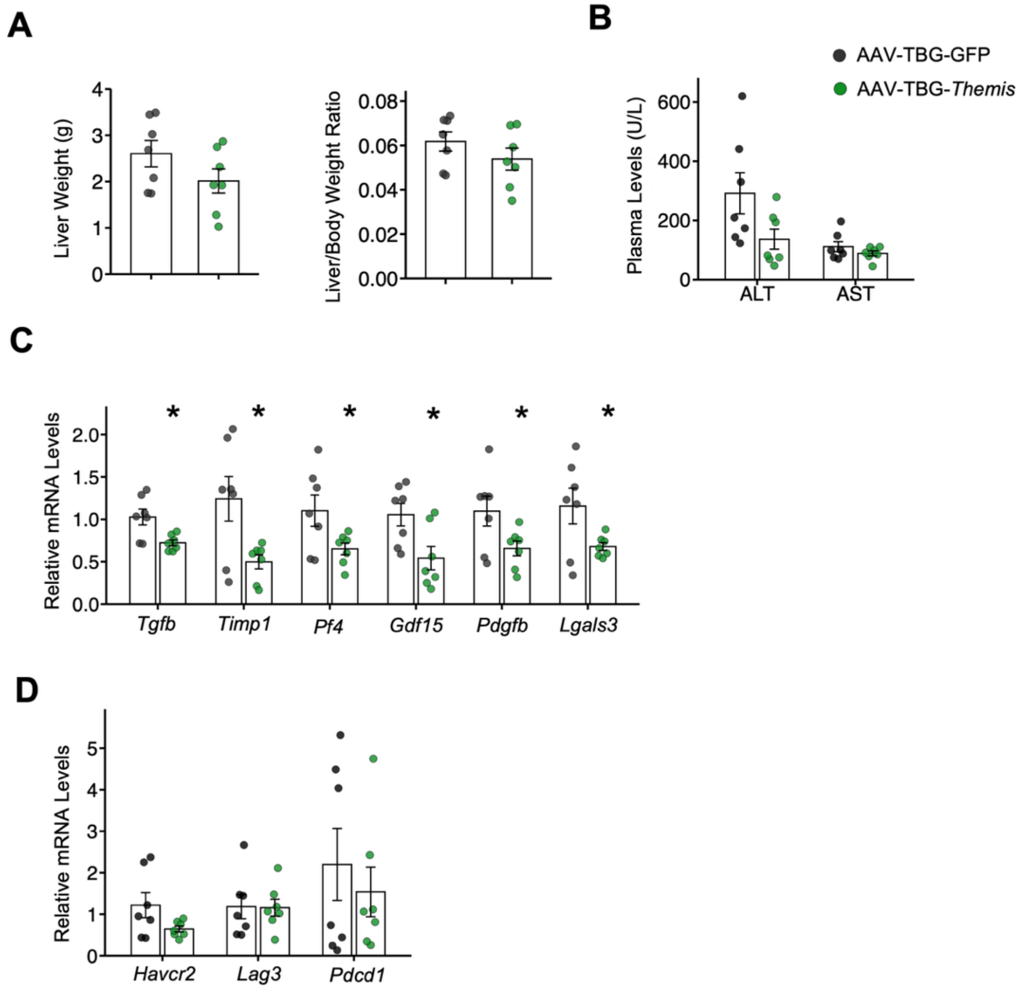

Supplementary Figure 10. AAV-mediated THEMIS overexpression in the liver. Male C57BL/6 mice were fed on a MASH diet for 14 weeks when mice were tail-vein injected with either AAV-TBG-GFP (n = 7) or AAV-TBG-Themis (n = 7). Mice were continuously fed on a MASH diet for another 6 weeks. (A) Tissue weight and liver/body weight ratio. (B) Plasma ALT and AST levels. (C-D) qPCR analysis of gene expression. Values represent mean ± SEM; two-tailed unpaired Student's *t* test. ∗ $P < 0.05$.

Supplementary Table 1

Senescence	De novo lipogenesis	Fatty acid oxidation	Proliferation
Cxcr2	Acly	Acadl	Mki67
Ptges	Acsl3	Crat	Pcna
Itpka	Hsd17b12	Etfdh	Top2a
Il1a	Acss2	Hadh	Mcm2
Il1b	Elovl6	Acadm	Mcm3
Il7	Acacb	Cpt2	Mcm4
Il2	Hacd3	Crot	Mcm5
Fgf2	Elovl5	Hadha	Mcm6
Csf1	Srebf1	Hadhb	Mcm7
Gem	Acaca	Acox3	Ccna2
Tnfrsf1b	Fads6	Acads	Ccnb2
Selplg	Fasn	Acad10	Ccnd1
Serpine1	Elovl2	Fabp1	Ccnd2
Cd9	Hacd2	Etfb	Ccnd3
Spx	Fads2	Echs1	Cdk2
Plat	Fads1	Gcdh	Cdk4
Gdf15	Scd1	Acat1	Cdk6
Mmp2		Etfa	Tyms
Mmp13		Acad11	Rrm1
Mmp12		Acaa1a	Rrm2
Icam1		Acadvl	Ung
Sema3f		Acox2	Fen1
Mif		Ehhadh	Prim1
Igfbp3		Eci1	Pole
Cxcl16		Hsd17b4	Polq
Ccl2		Acaa2	Cdc6
Ccl7			Cenpf
Ccl5			Cenpe
Ccl4			Cenpa
Timp2			Aurka
Bmp6			Kif11
Edn1			Kif23
Plau			Bub1b
Ptger2			Mad2l1
Mmp14			Ndc80
Angpt1			Spc25
Tnfrsf11b			Cenpn
Acvr1b			Cenpk
Ets2			Hist1h1b
Cdkn1a			Ube2s
Areg			Anapc1
			Anapc5
