# Supplementary material for: THEMIS attenuates MASH by suppressing disease-associated hepatocyte induction and hepatocyte senescence in mice
Source: J Clin Invest. 2026 May 1;136(9):e199303. doi: 10.1172/JCI199303 (PMC13132366; doi:10.1172/JCI199303)

Figure 1G

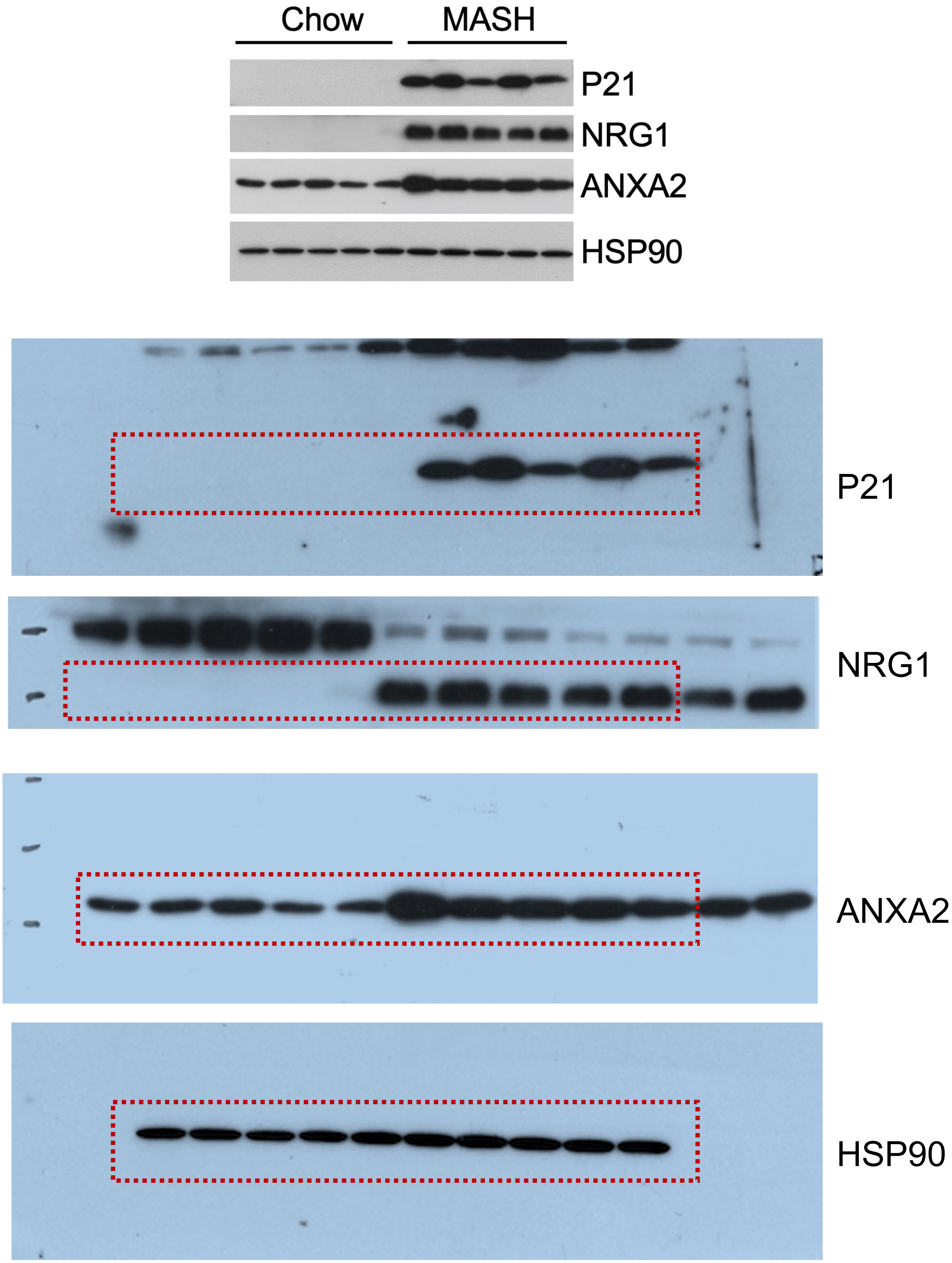

Figure 2D

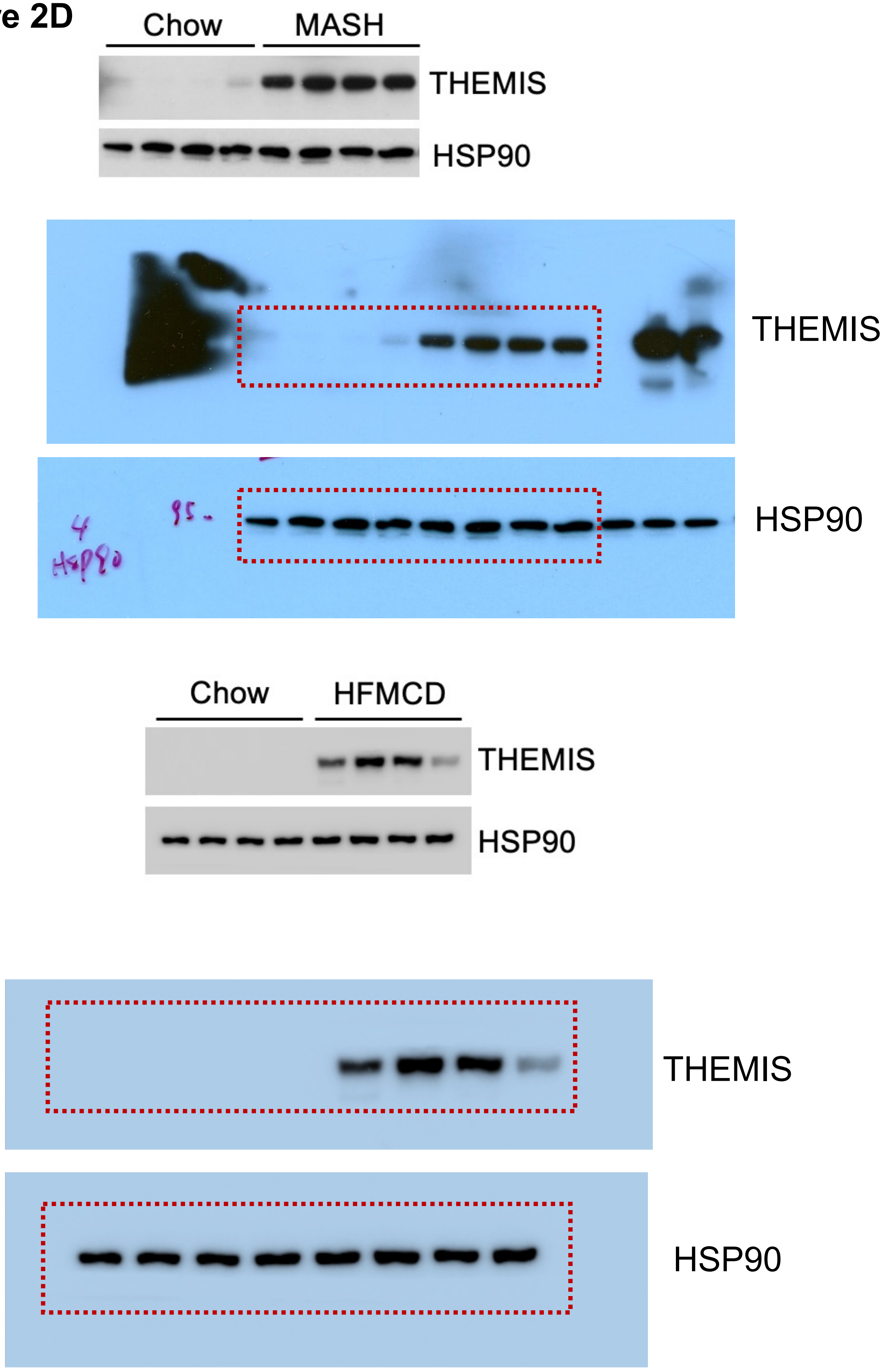

Chow
MASH
THEMIS
HSP90
THEMIS
HSP90
Chow
HFMCD
THEMIS
HSP90
THEMIS
HSP90

Figure 2J

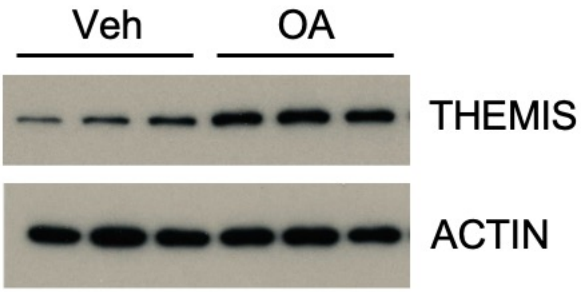

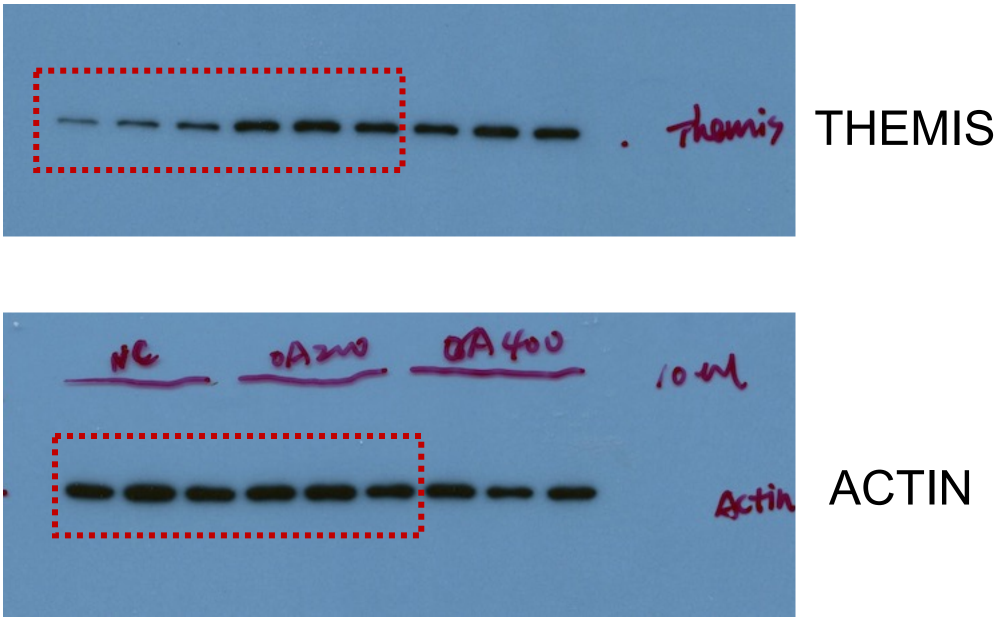

Figure 3H

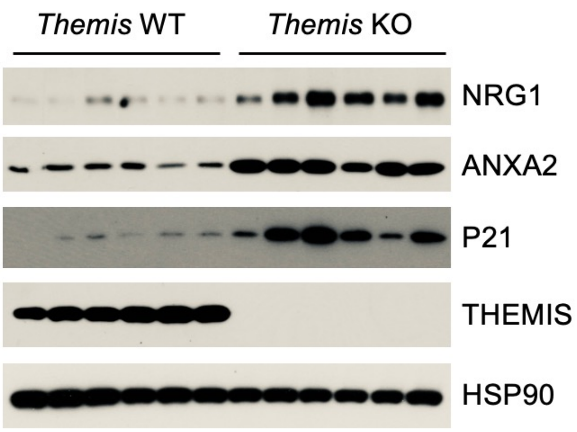
Themis WT
Themis KO
NRG1
ANXA2
P21
THEMIS
HSP90

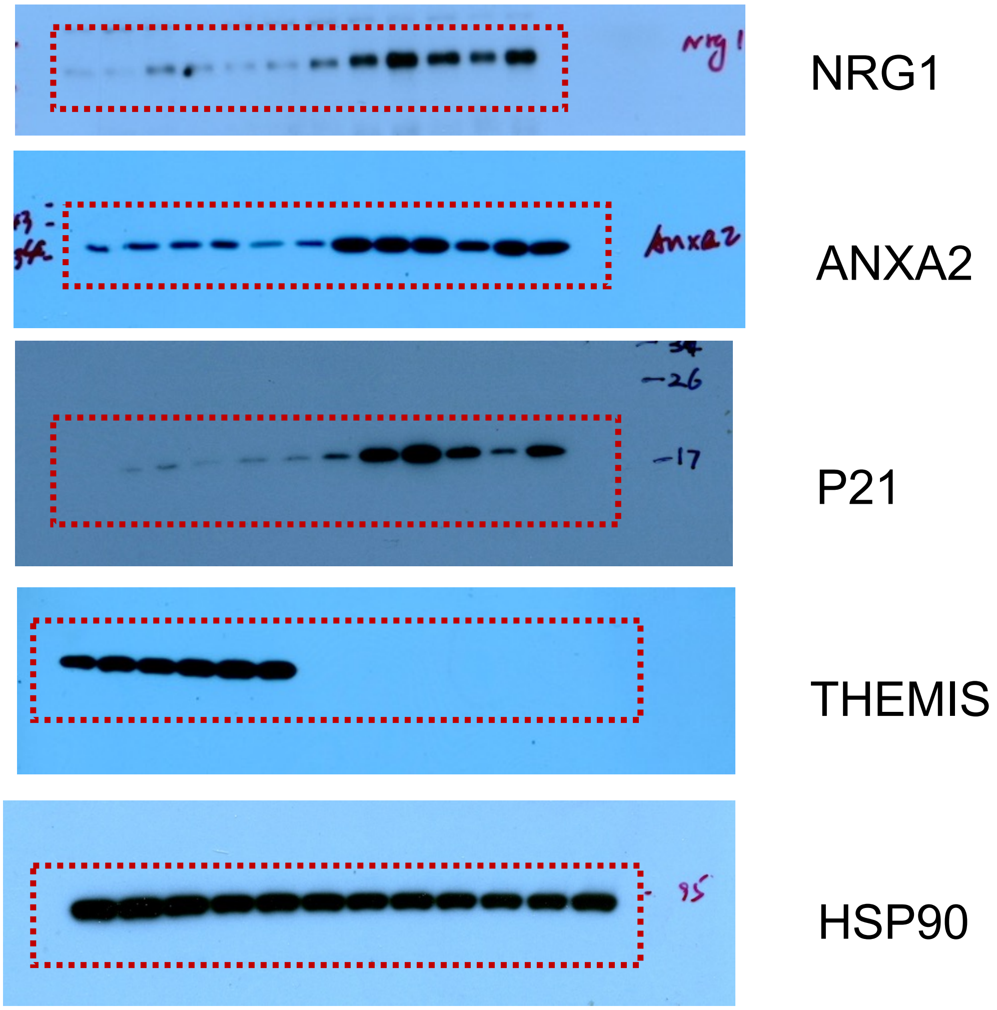
NRG1
ANXA2
P21
THEMIS
HSP90

Figure 4F

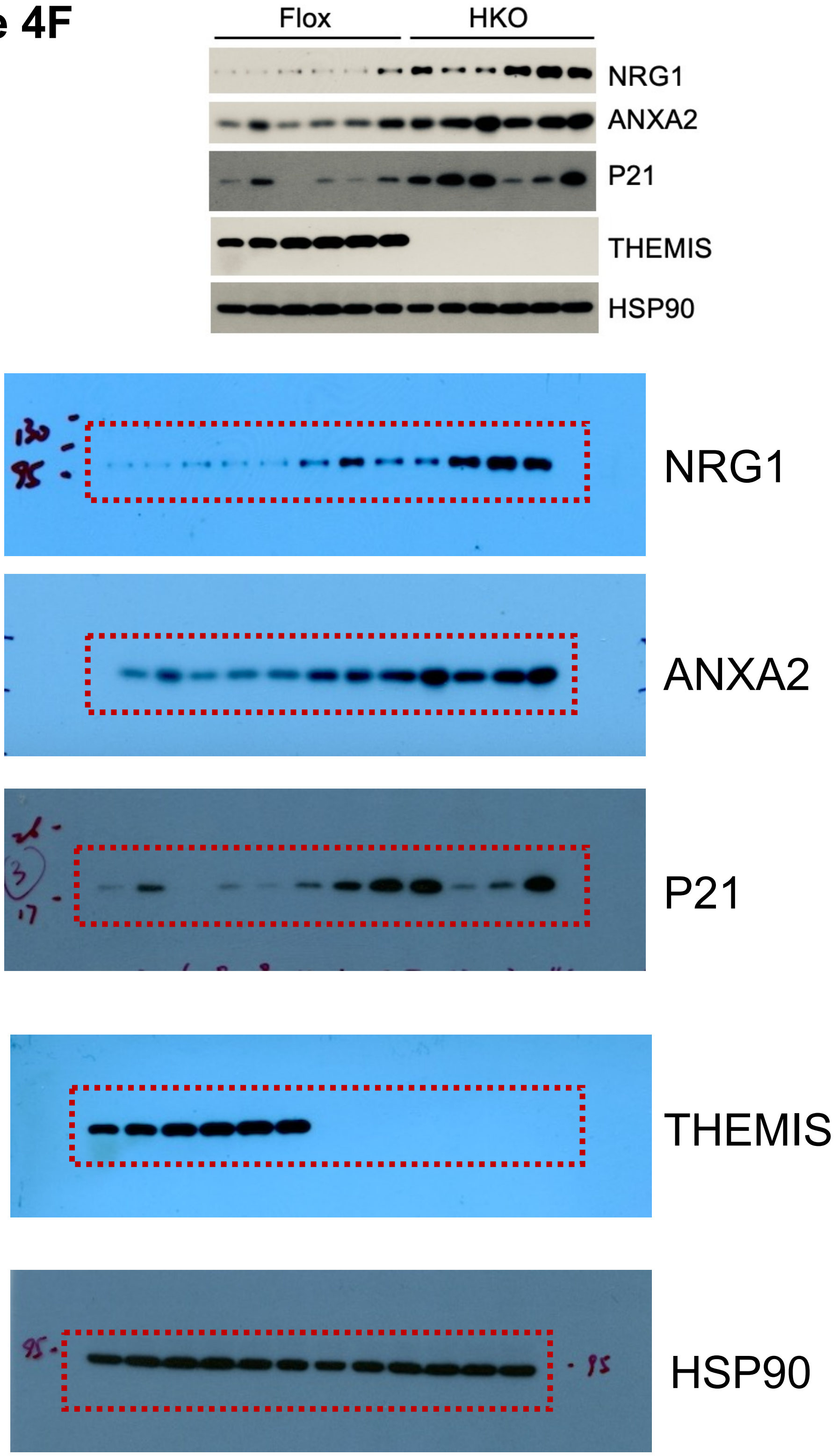

Flox
HKO
NRG1
ANXA2
P21
THEMIS
HSP90
NRG1
ANXA2
P21
THEMIS
HSP90

Figure 6A

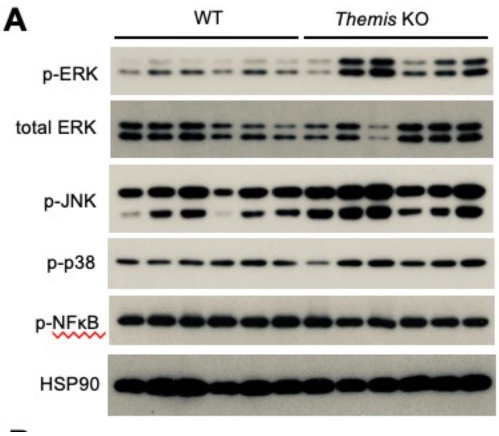

A
WT
Themis KO
p-ERK
total ERK
p-JNK
p-p38
p-NFκB
HSP90

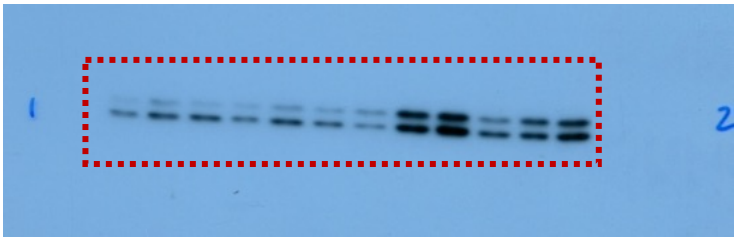

p-ERK

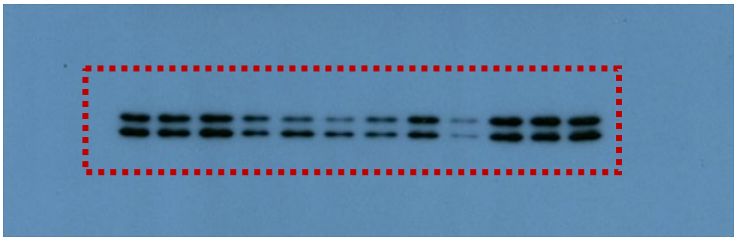

total-ERK

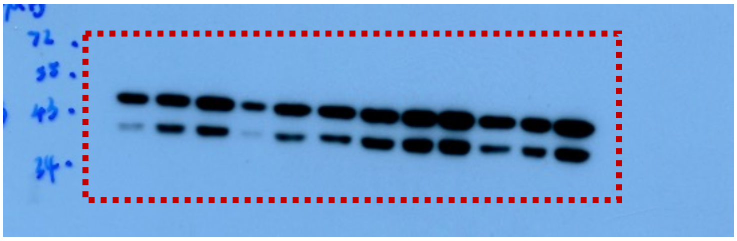

p-JNK

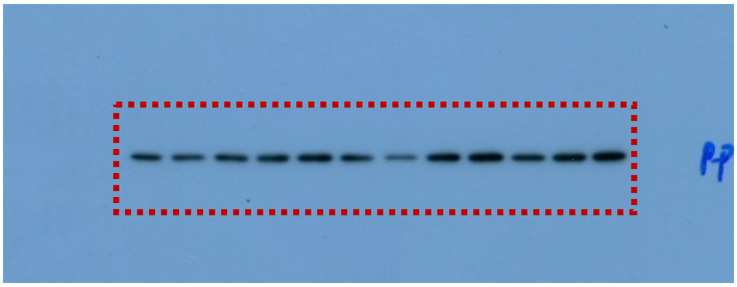

p-p38

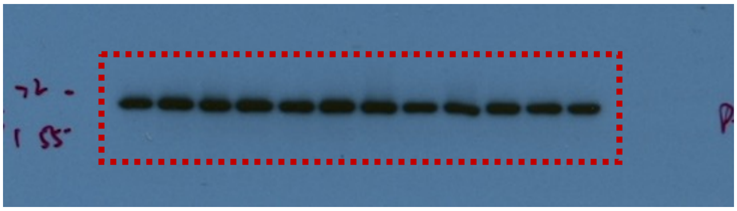

p-NFκB

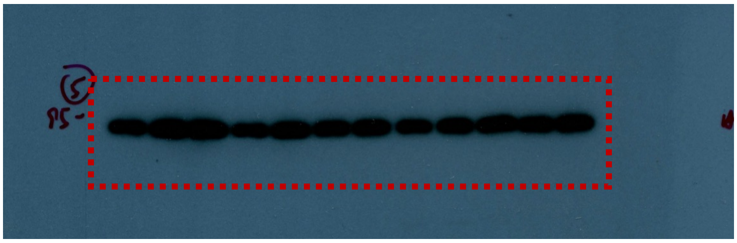

HSP90

Figure 6B

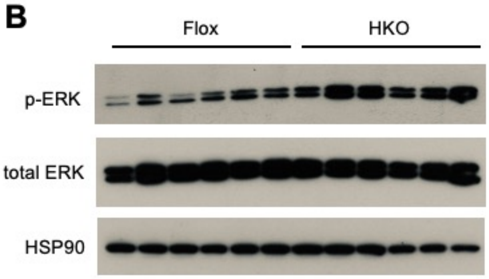

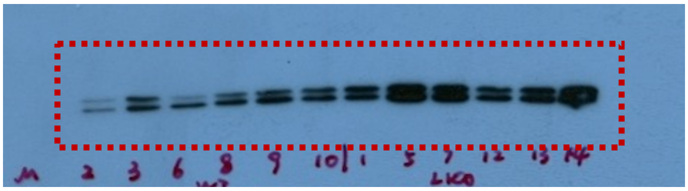

p-ERK

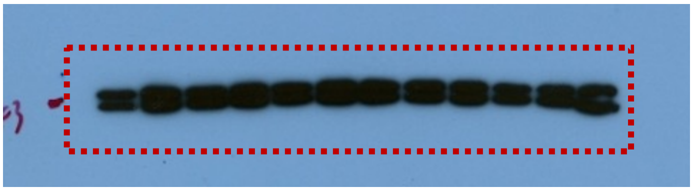

total-ERK

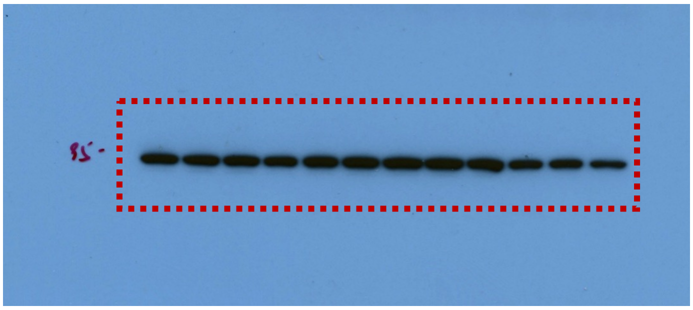

HSP90

Figure 6C

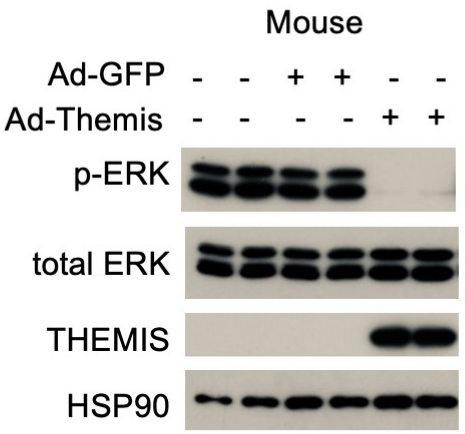

Mouse
Ad-GFP - - + + - -
Ad-Themis - - - - + +
p-ERK
total ERK
THEMIS
HSP90

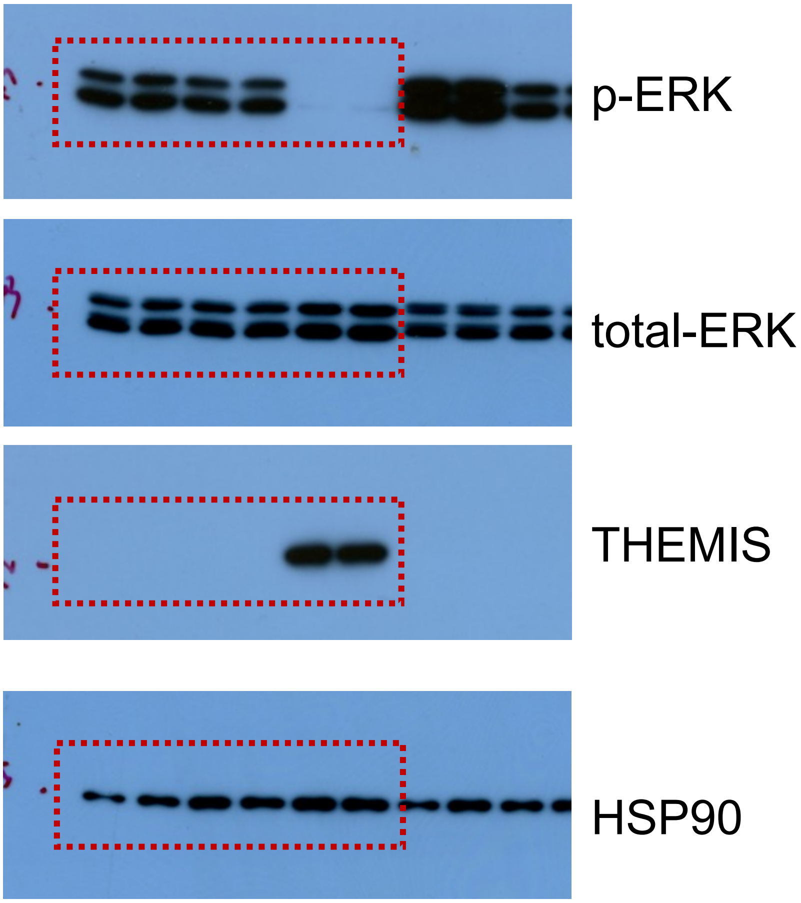

p-ERK
total-ERK
THEMIS
HSP90

Figure 6C continued

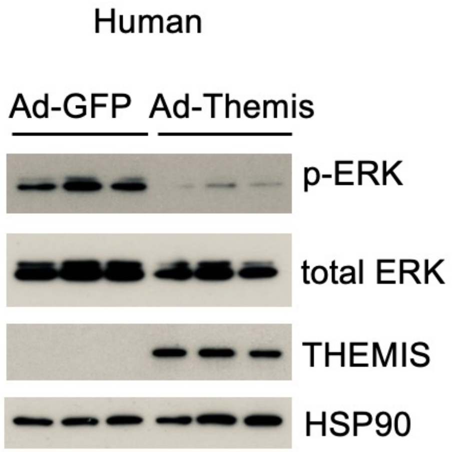

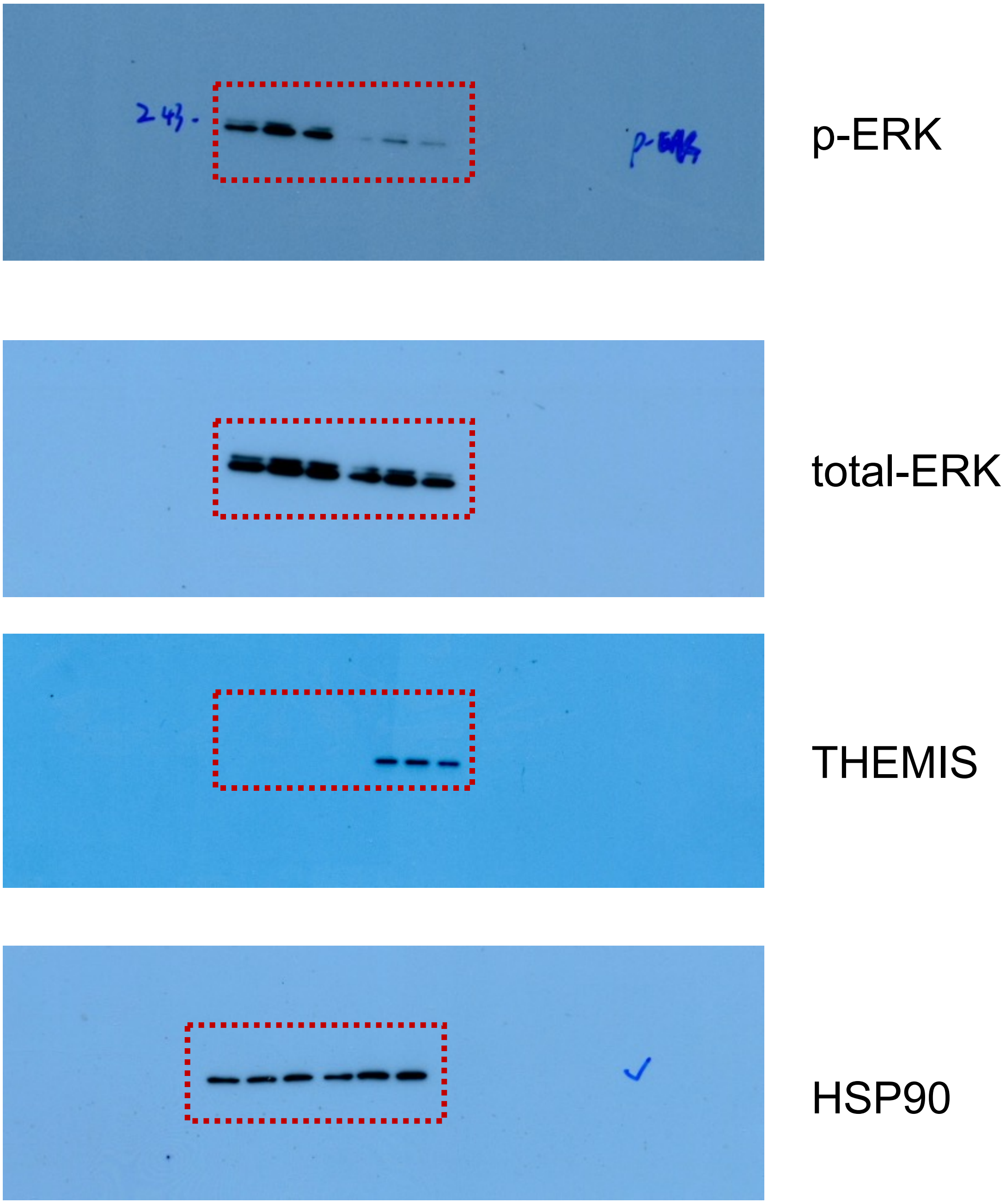

Figure 6D

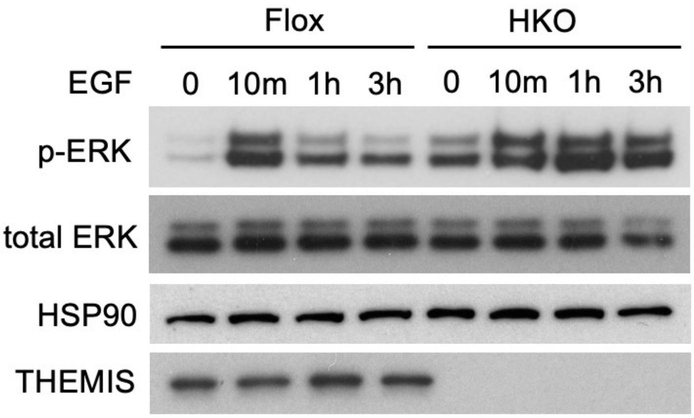

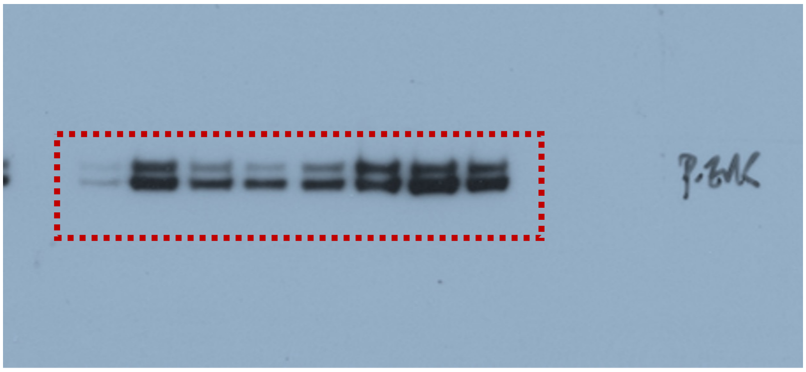

p-ERK

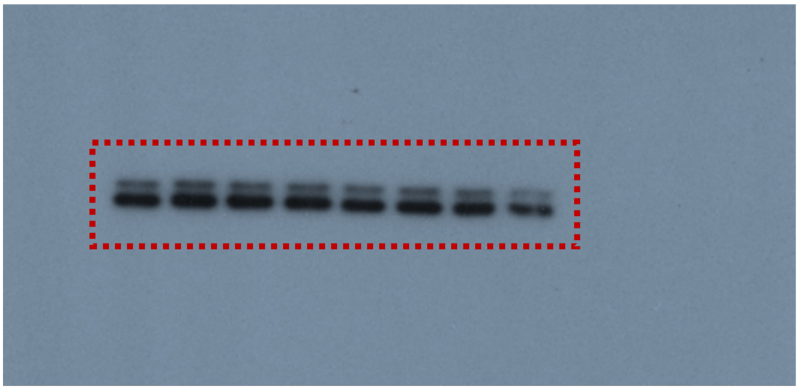

Total-ERK

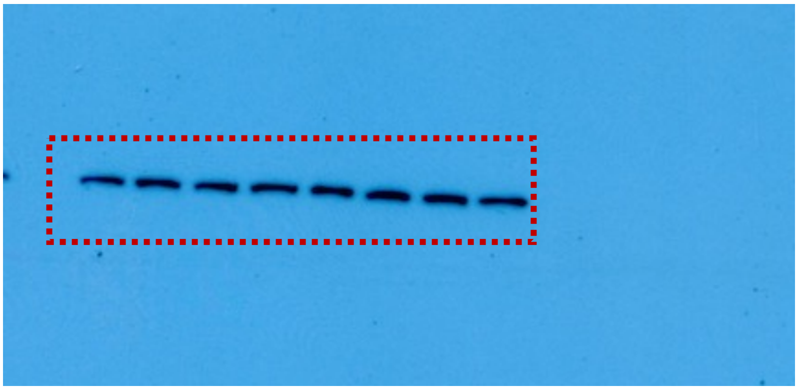

HSP90

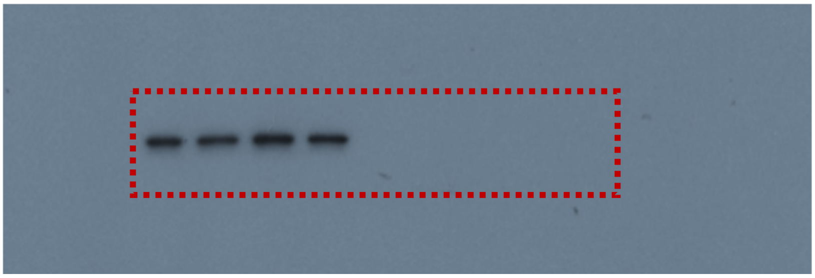

THEMIS

Figure 6E

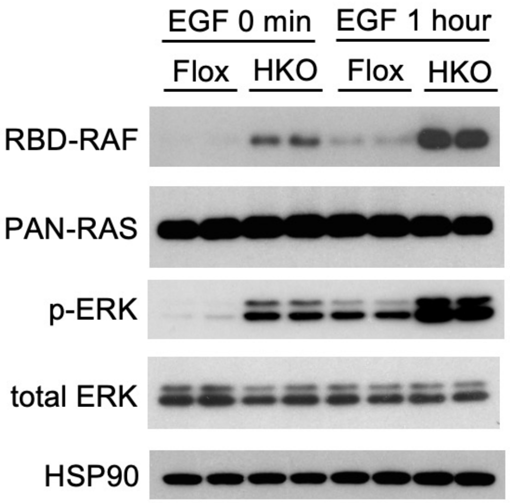
EGF 0 min
EGF 1 hour
Flox
HKO
Flox
HKO
RBD-RAF
PAN-RAS
p-ERK
total ERK
HSP90

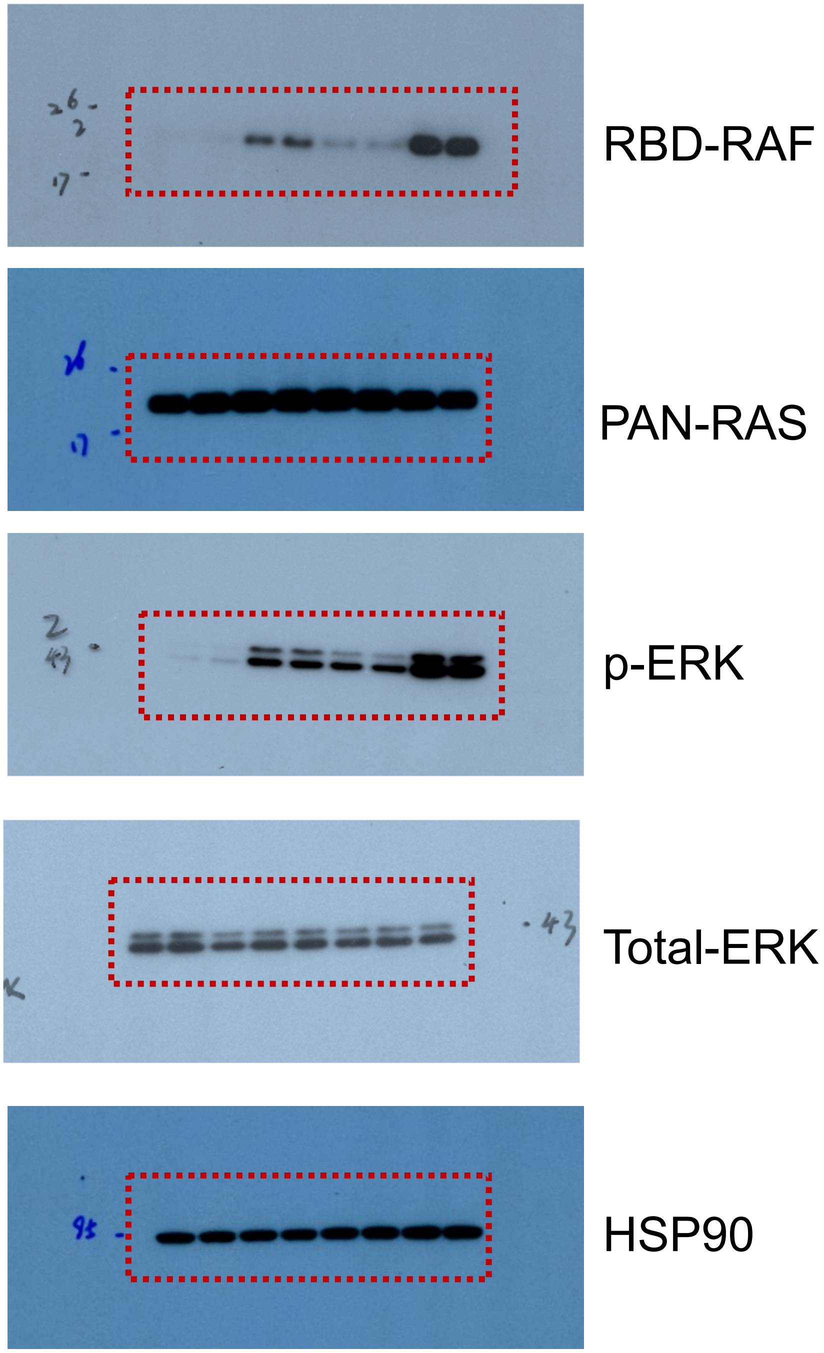
RBD-RAF
PAN-RAS
p-ERK
Total-ERK
HSP90

Figure 6G

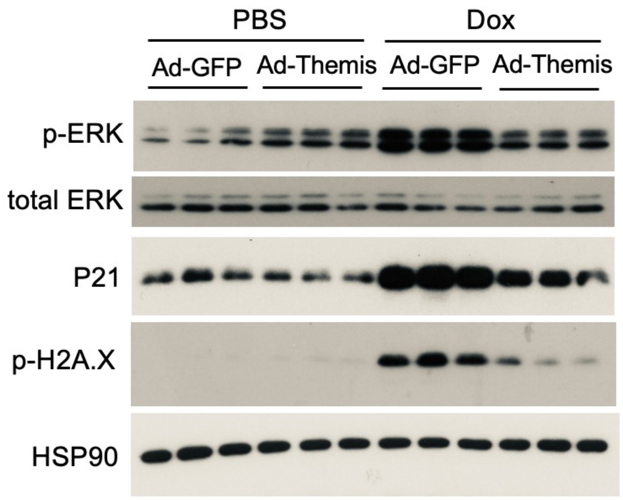
PBS
Dox
Ad-GFP
Ad-Themis
Ad-GFP
Ad-Themis
p-ERK
total ERK
P21
p-H2A.X
HSP90

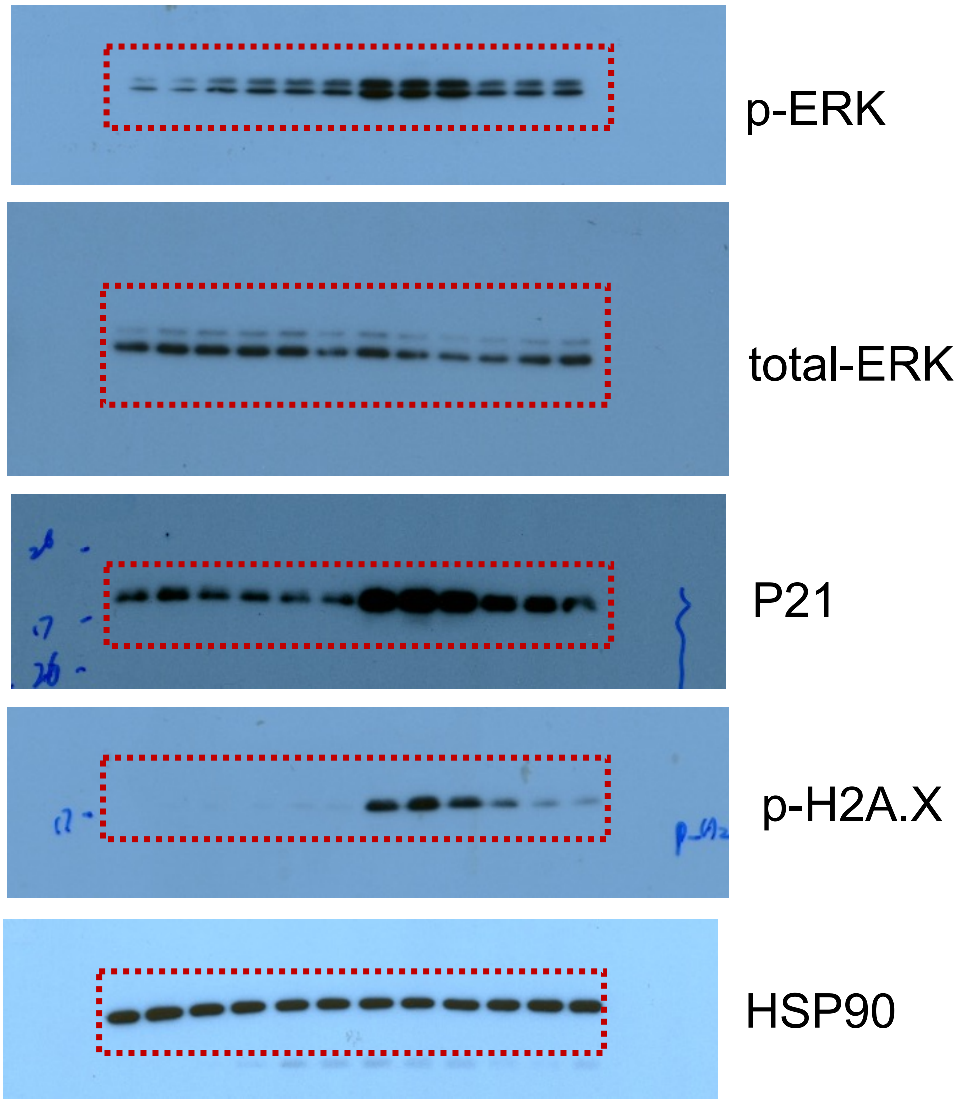
p-ERK
total-ERK
P21
p-H2A.X
HSP90

Figure 6H

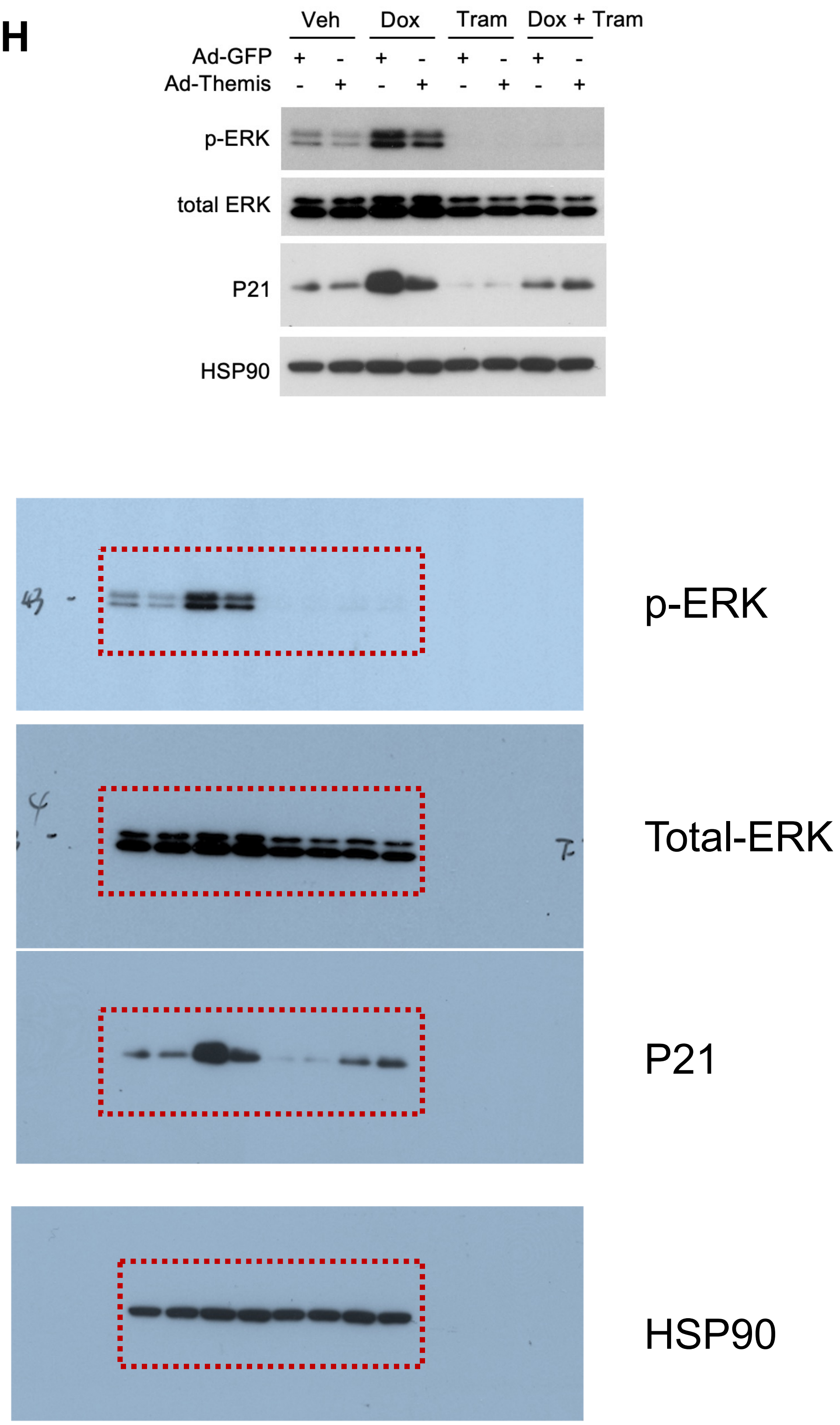

Veh
Dox
Tram
Dox + Tram
Ad-GFP + - + - + - + -
Ad-Themis - + - + - + - +
p-ERK
total ERK
P21
HSP90
p-ERK
Total-ERK
P21
HSP90

Figure 7E

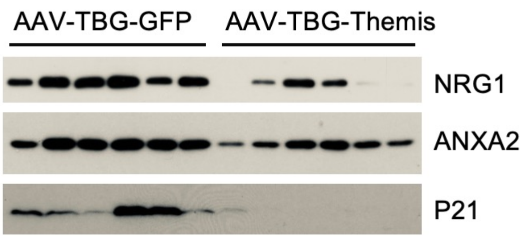

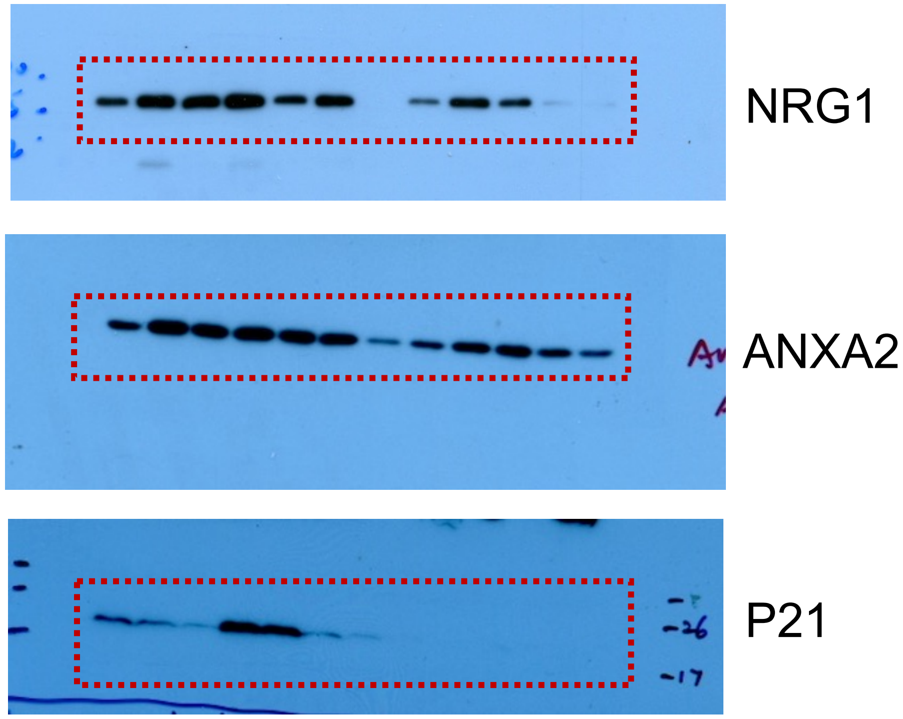

Figure 7E continued

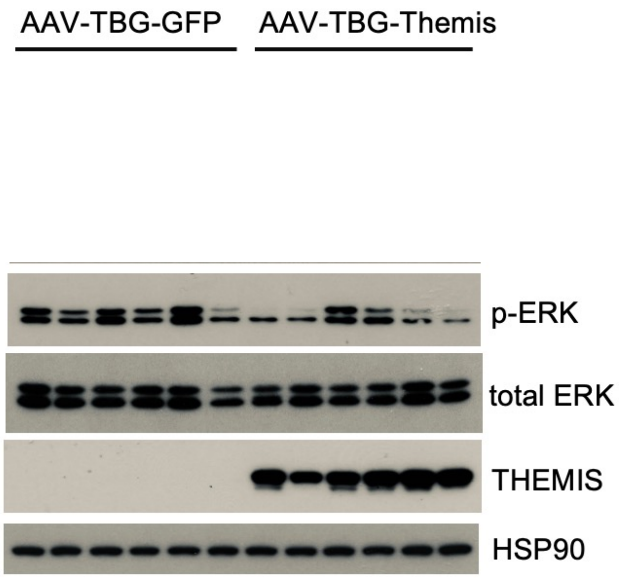

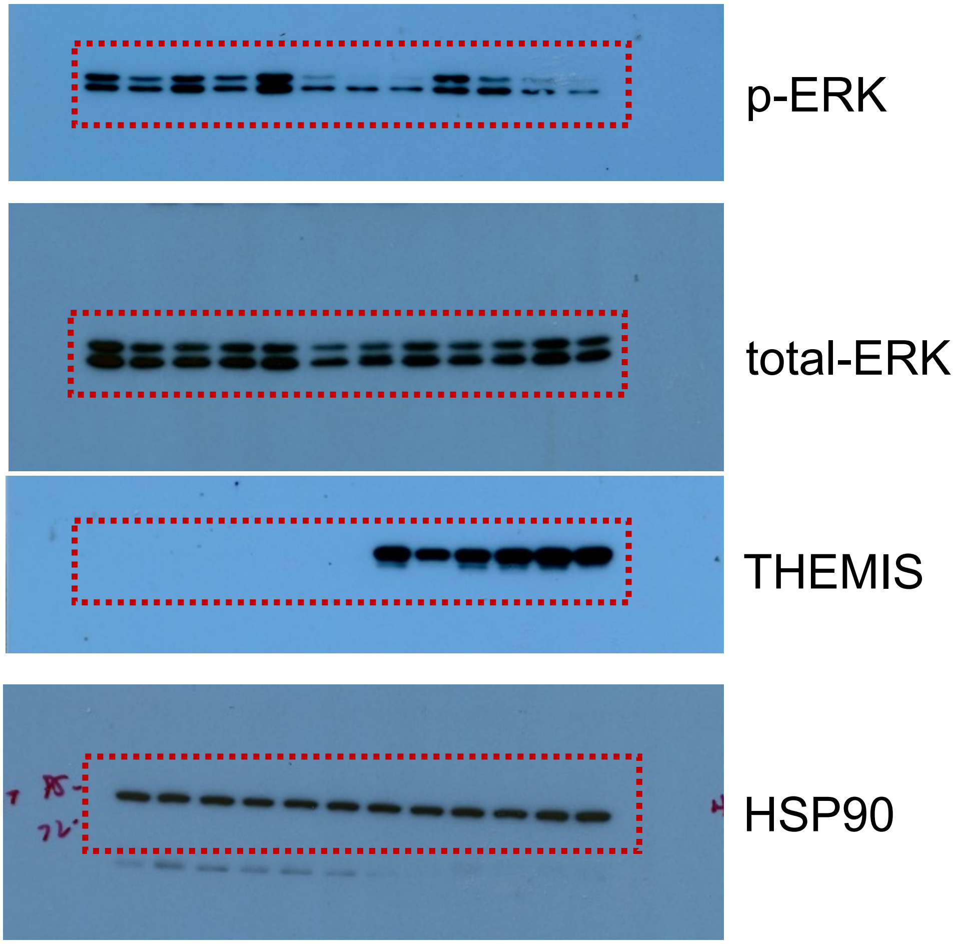

Figure 8G

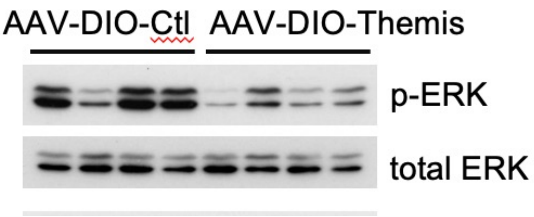

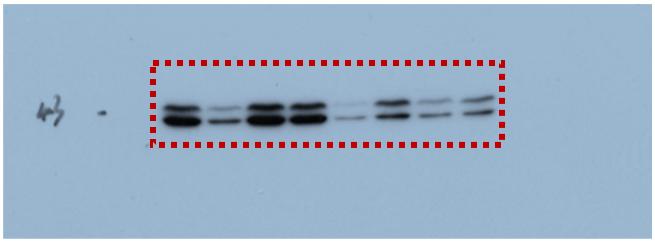

p-ERK

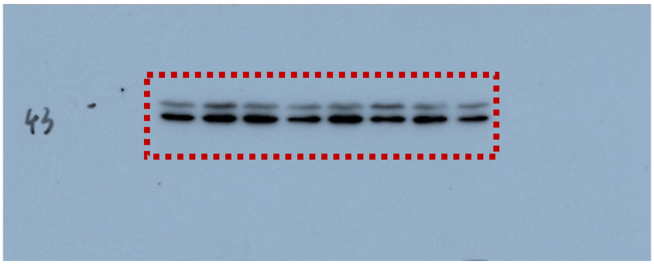

total-ERK

Figure 8G continued

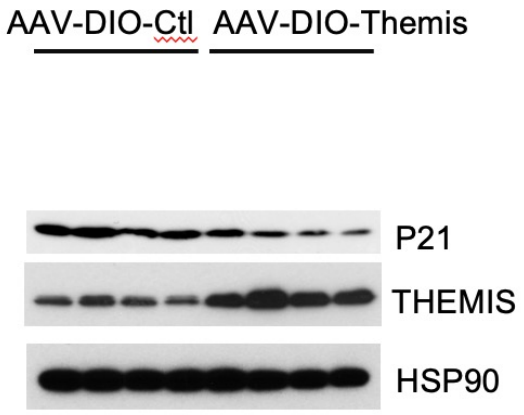

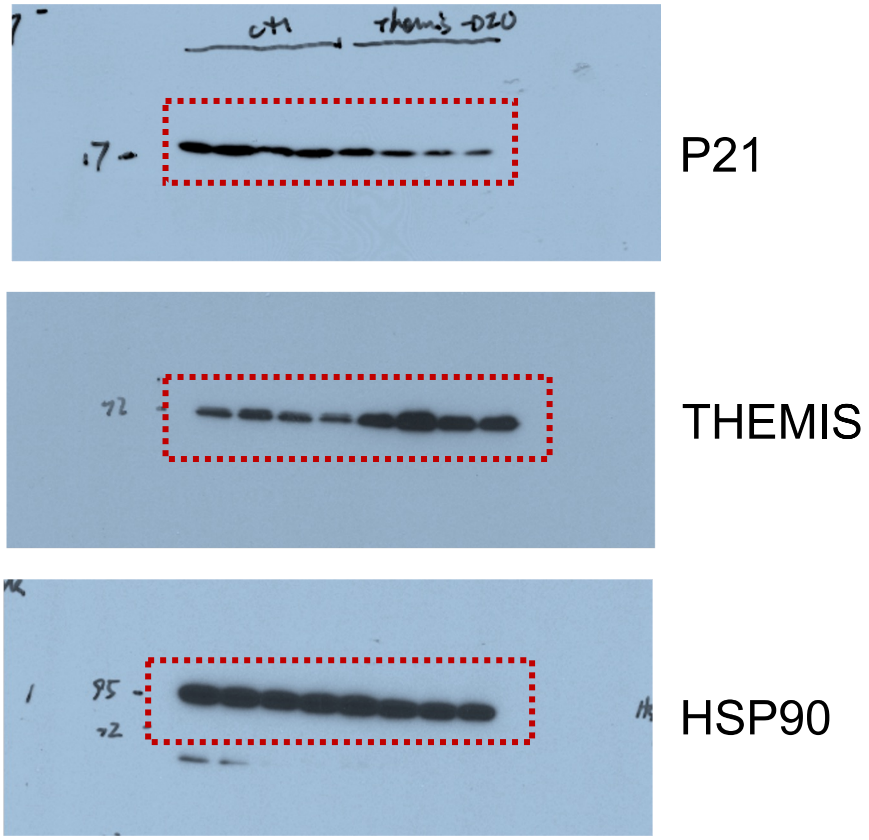

Supplementary Figure 9C

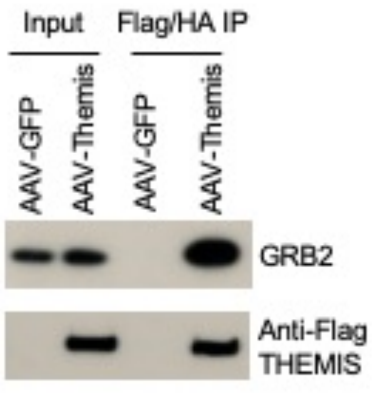

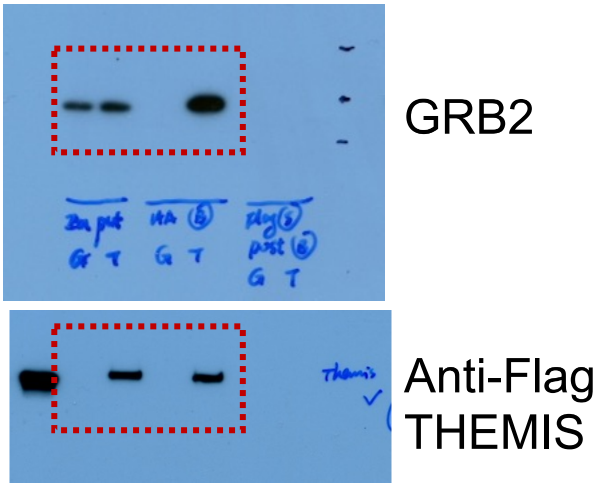

Supplementary Figure 9D

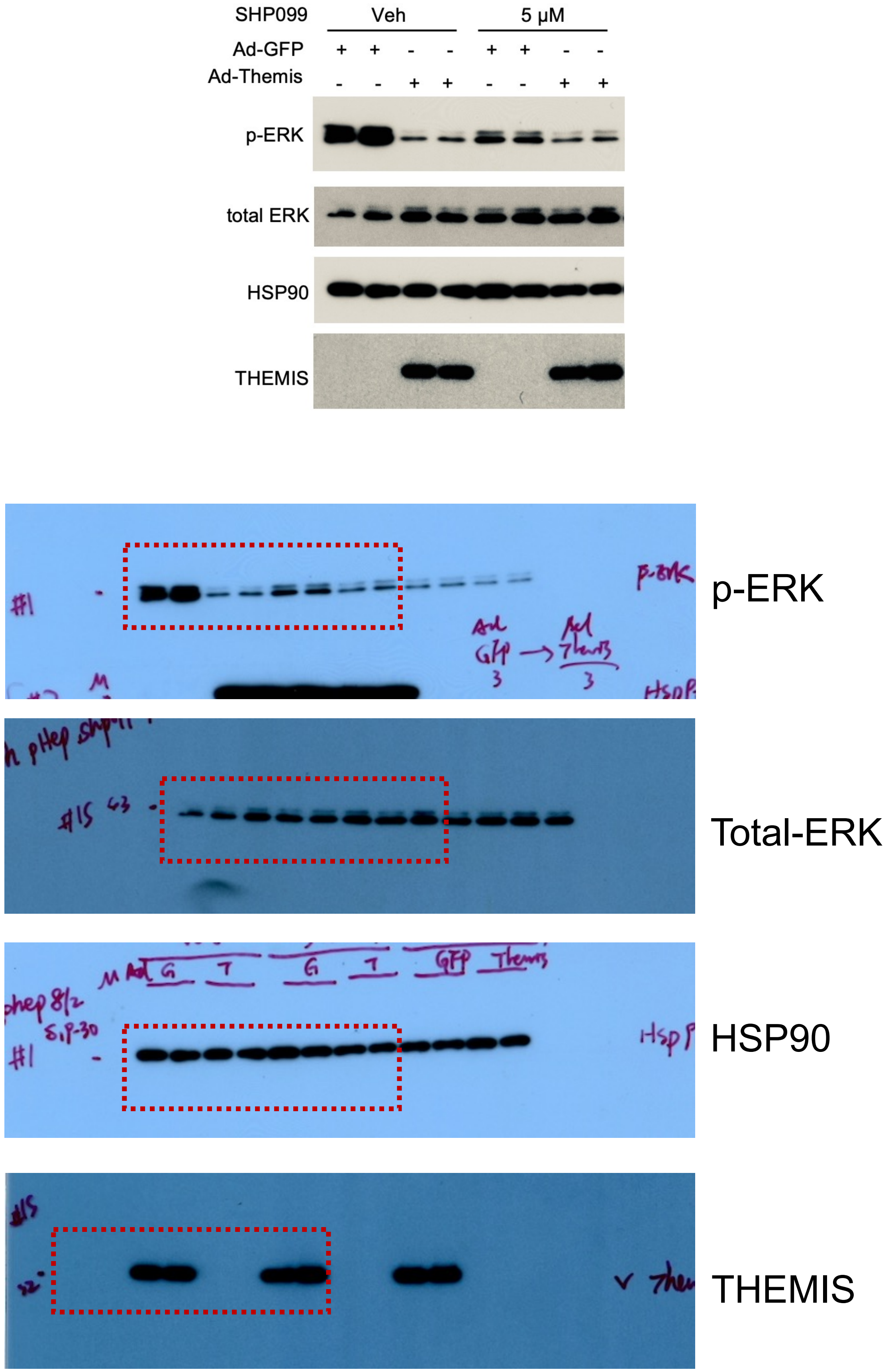

Supplement: Unedited blot and gel images [file jci-136-199303-s136.pdf]
